# VIII World Rett Syndrome Congress & Symposium of rare diseases, Kazan, Russia

**DOI:** 10.1186/s13039-018-0412-2

**Published:** 2018-12-24

**Authors:** Ivan Y. Iourov, Svetlana G. Vorsanova, Yuri B. Yurov, Thomas Bertrand

**Affiliations:** 1 Mental Health Research Center, 117152 Moscow, Russia; 2 Veltischev Research and Clinical Institute for Pediatrics of the Pirogov Russian National Research Medical University, Ministry of Health of Russian Federation, 125412 Moscow, Russia; 3 Department of Medical Genetics, Russian Medical Academy of Continuous Professional Education, Moscow, 125993 Russia; 4 Rett Syndrome Europe, https://www.rettsyndrome.eu/

**Keywords:** Chromosome X, Rare disorders, Rare chromosome disorders, Rett syndrome, Translational medical research

## Abstract

**Background:**

VIII World Rett Syndrome Congress & Symposium of Rare Diseases was held in Kazan, Russia from 13 to 17 May 2016. Although it has been a while since the event, specific problems highlighted by the contributors to the scientific program have stood the test of time. The Symposium of Rare Diseases has shown that studying Rett syndrome provides clues on molecular and cellular mechanisms for a variety of rare genetic/genomic disorders. Moreover, rare diseases associated with Rett-syndrome-like phenotype or *MECP2* mutations/copy number variations have been thoroughly covered by a number of contributors. In this respect, we have found that a review dedicated to the scientific program of the VIII World Rett Syndrome Congress & Symposium of Rare Diseases could be an important addition to current literature.

**Conclusion:**

Taking the opportunity to review the World Rett Syndrome Congress & Symposium of Rare Diseases at Kazan, we have made an attempt to describe a number of achievements and developments in the field of studying Rett syndrome and rare diseases in Russia. Furthermore, chromosomal abnormalities/disorders have been considered in the rare disease context. Such approach to chromosomal abnormalities/disorders has been found to be rather new for an appreciable part of international researchers and health care providers. We do hope that this congress review may be helpful not only for those who are interested in local development of research and management of rare genetic disorders, but also for international researchers and clinical community of rare disease specialists.

**Electronic supplementary material:**

The online version of this article (10.1186/s13039-018-0412-2) contains supplementary material, which is available to authorized users.

## Introduction

According to Kathy Hunter, the founder of International Rett Syndrome (OMIM: 312750; RTT) Association in the United States of America, seven previous World Congresses dedicated to RTT have been held in Antwerp (Belgium), Gothenburg (Sweden), Edinburgh (Scotland), Karuizawa (Japan), Helsinki (Finland), Paris (France), New Orleans (USA). The millennium city of Kazan, the capital of Tatarstan Republic of the Russian Federation, has accommodated the eighth congress, which has been acknowledged as a success [1]. Indeed, 686 participants from 21 countries have taken part in the program of the congress.

Russian RTT Association (NGO “Association for assistance to Rett syndrome patients” chaired by Olga V. Timutsa) http://rettsyndrome.ru has made significant efforts to organize this long-awaited meeting between international scientists, doctors, families and RTT girls. International scientific committee included the most recognized researchers of RTT: Angus Clarke (Wales, UK), Alan Percy (Birmingham, USA), Alessandra Renieri (Siena, Italy), Eric E.J. Smeets (Maastricht, the Netherlands), Helen Leonard (Perth, Australia), Laurent Villard (Marseille, France), Leopold M.G. Curfs (Maastricht, the Netherlands), Meir Lotan (Ariel, Israel), Sakkubai Naidu (Baltimore, USA), Svetlana G. Vorsanova (Moscow, Russia), Thomas Bertrand (Antony, France), Yoshiko Nomura (Tokyo, Japan), Yuri B. Yurov (Moscow, Russia); head of the scientific program committee — Ivan Y. Iourov (Moscow, Russia). The group of expert researchers has developed a basis for the scientific success of the VIII World Rett Syndrome Congress. Additionally, the event has included the Symposium of Rare Diseases, which has allowed to attain even more success and has contributed to the development of rare disease research in Russia. Here, we have attempted to review scientific and practical aspects of the great body of research on RTT and rare diseases presented at the VIII World Rett Syndrome Congress & Symposium of Rare Diseases in the historical perspective. This information is likely to be valuable both for those who are interested in local development of research and management of rare genetic disorders and for international researchers and clinical community of rare disease specialists.

## RTT and *MECP2*-related diseases

In 1966, RTT phenotype was first reported by Andreas Rett, an Austrian pediatric neurologist from Vienna [2]. It took then about 17 years before Bengt Hagberg, a Swedish child neurologist, together with Jean Aicardi, Karin Dias, and Ovidio Ramos, published their landmark article demonstrating RTT to be the leading cause of dramatic intellectual disability among girls [3]. Another 16 years passed before the genetic cause of RTT was discovered [4]. As we know now, *MECP2* gene mutations are responsible for the majority of classic RTT cases. This genetic defect is the main target of molecular RTT diagnosis. However, RTT diagnosis is still based on clinical findings fitting diagnostic criteria [5]. During the last two decades, the body of RTT research has grown extensively forming theoretical and practical basis for helping individuals with RTT and associated neurodevelopmental disorders [6–14]. To gain more information on RTT phenotypes and clinical course, one can address the aforecited thorough and timely reviews.

In parallel, *MECP2* biology has been another focus of RTT research [15–17]. These studies have not only described the consequences of alterations to *MECP2-*related pathways [15–17], but also uncovered a variety of neurological and psychiatric disorders caused by *MECP2* mutations [10–14]. Interestingly, *MECP2* losses (Xq28 microdeletions) are likely to be associated with mild classical and atypical subtypes of the RTT and are relatively common in RTT females without pathogenic *MECP2* sequence variations [18, 19]. Further studies of *MECP2*-mutation-negative patients from RTT cohorts have demonstrated that RTT or RTT-like phenotypes can be a result of CNV (copy number variations) and mutations affecting genes other than *MECP2* [20–23]. Consequently, RTT phenotype seems to be more genetically heterogeneous than generally recognized. Accordingly, a hypothesis suggesting that the only way of comprehensive pathway-based analysis of a disease phenotype is to address a broad spectrum of genome pathology (CNV, monogenic mutations, genome/chromosome instability, chromosomal abnormalities) and genetic-environmental interactions [24] is applicable for complex neuropsychiatric disorders and brain diseases associated with mutations affecting regulatory genes (e.g. RTT).

Molecular studies of RTT and RTT-like phenotypes have shown several tens of genes associated with these devastating conditions [25, 26]. Accordingly, such genes are likely to be involved in a generalized pathway altered in RTT and phenotypically resembling conditions. The definition of this pathway may be used for the development of new therapeutic strategies for this broad spectrum of brain pathology [27]. In conclusion, it is to be emphasized that RTT studies are able to provide further insights to rare genetic diseases, as well. Therefore, one should not be surprised that the VIII World RTT Congress has accommodated the Symposium of Rare Diseases.

## RETT syndrome research in Russia

It is reasonable to ask: “Why Russia?” To answer this question, it appears important to address RTT research in Russia. The first articles reporting studies of Russian RTT cohort were published in the nineties of the last century [28, 29]. Some years later, Russian RTT cohort was expanded to 57 girls and three boys and was investigated by molecular genetic and molecular cytogenetic techniques [30]. Shortly after, *MECP2* mutation analysis of Russian RTT cohort was published [31]. Another major success of Russian RTT research has been associated with the World Congress on RTT 2000 held in Karuizawa, Japan. There, a body of numerous achievements in RTT research made by Russian specialists has been reported [30, 32–37]. More specific achievements in Russian RTT research have been as follows: uncovering the 2.5% RTT incidence in institutionalized mentally retarded girls [38], RTT-specific epigenetic features (i.e. disturbance in the sequence of replication/synchronous replication of the X chromosome loci in both mutation-positive and mutation-negative RTT females) [30, 36–40]; genotype/phenotype correlations in Russian RTT cohort associated with parental-like effect on X chromosome inactivation skewing [31, 40]; uncovering CNV and microdeletions at Xq28 and other chromosomal loci causing RTT and RTT-like phenotypes [12–20]; reports on somatic chromosomal mosaicism to underlie rare cases of RTT in boys [29, 30]. It is to note, that studying RTT and *MECP2* mutations in boys has long been a focus of genetic studies of Russian RTT cohort [29, 30, 41]. Actually, there is still a need to continue these studies for uncovering new rare genetic diseases associated with *MECP2* mutations/CNV [12, 42, 43]. Probably the best example of such a disease is *MECP2* duplication syndrome, which phenotype is drastically different from RTT [42, 43]. Recently, a number of patients with *MECP2* duplication syndrome from the Russian cohort of children with intellectual disability, autism, epilepsy and congenital malformations have been described [44]. Although Russian RTT cohort includes more than 350 patients, it seems that clinical/molecular diagnosis of RTT has not still achieved the level required to estimate properly the incidence of the disease in all Russia’s regions. Accordingly, we have suggested that the World Congress on RTT can be a starting point to stimulate RTT research all across Russia. As can be seen from the above, there is a strong RTT research background in Russia and demand for creating a milieu for a successful scientific event dedicated to RTT and rare diseases.

## Scientific program

The scientific program included a plenary session, open lectures and scientific symposium during the first day, then parallel sessions were conducted on the following days. The parents along with their children had an opportunity to visit rehabilitation centers, receive consultations from international health-care professionals and visit lectures dedicated to scientific and/or therapeutical aspects of the disease. During the plenary session, an overview of the situation in Russia was made. Russian RTT cohort includes 391 girls and 3 RTT boys. Most of the girls have *MECP2* mutations. However, amongst the 29% of mutation-negative RTT girls, a vast majority were found to have microdeletions at Xq28 (including *MECP2)*. Data on “MECP2 interactome” have been presented in the context of possible drug targets and genotype/phenotype correlations. Systems biology approaches to RTT have been discussed.

Alan Percy’s video lecture overviewed the natural history of RTT since 2003. Dr. Helen Leonard gave a clear explanation of the role of databases and population-based registries in understanding the disease. Examples of studies were given such as the symptoms’ severity of girls and women with a C-terminal deletion of *MECP2* compared to those who have point mutations in this gene, or breathing patterns and other autonomic functions and their relationship with age or mutation type. Professor Naidu’s talk focused on the hypothesis that glutamate toxicity could be the causal effect of seizures in RTT. Indeed, the patients lack glutamate receptor at the post-synaptic level. Consequently, the uncaptured glutamate molecules bind to another type of receptors at the extra-synaptic level, causing toxic effects.

Professor Zoghbi has revisited recent therapeutic possibilities through a video lecture. One idea is to try to boost the expression of the protein to counter-balance the loss of efficacy of the mutated protein. This would lead to finding other proteins that up-regulate *MECP2* expression. Another approach is to work on different types of neurons of different brain areas because some might be more important for seizure control. The third, and probably most novel approach, is the electric deep-brain stimulation of neuron bundles in hippocampus. A new study has showed that, in murine models, a benefit in the memory and behavior is clearly observed. The next studies need to focus on the frequency and length of stimulations needed to keep a beneficial effect. Moreover, the study needs to check whether the beneficial effect could be durable.

Sarojini Budden has explained that gallbladder pain occurs more frequently in individuals with RTT as to general population. It has been observed that children, who are very active at a younger age, are likely to become depressed when they are more restricted in movements at an older age. In another talk, she has reminded that RTT girls with early hypotonia without acquired sitting by 2 years seldom acquire walking. Rigidity begins often around 5–9 years; the first sign is the tightness of the heel cords. This can be related to a decreasing dopamine level in the brain. Drugs currently used for treating Parkinson’s disease might lead to improvements in RTT. One interesting observation has been related to the use of arm stretchers (elbow splints) to improve standing position and walking ability and to increase the focus on the action of walking.

A follow-up on the Maastricht Experience was given by Dr. E.E.J Smeets. He mentioned that there it is required to define the immaturity of the brainstem of each individual child and to identify the cardiorespiratory phenotype (forceful, feeble or apneutic breather). It is important to differ between seizures and non-seizures events. Some events as breath holding, hyperventilation, vacant spells, screaming laughter, stiffening, tremulousness, falling forward, pupil dilation, gazing and involuntary movements can be seen as seizures and they are not. An electroencephalography video monitoring can be helpful to differentiate seizures from non-seizure events.

A presentation of a new project “development of clinical guidelines for the management of communication in RTT” was given by Professor L.M.G Curfs. The project started during the European Rett Syndrome Conference in Maastricht in 2013. The project was conducted by Rett Expertise Centre Netherlands (Gill Townend and Leopold Curfs) with a core group: Helena Wandin (Sweden), Theresa Bastolotta (USA), Anna Urbanowicz (Australia), Sally-Ann Garrett (UK & Ireland).

Scientific symposia dedicated to rare diseases were chaired by members of scientific committee as well as Professors S.I. Kutsev (Moscow, Russia), V.I. Larionova (Saint Petersbourg, Russia), S.Y. Volgina (Kazan, Russia), Professor Dr. Thomas Liehr and others. The symposia showed that studying RTT provides clues on molecular and cellular mechanisms for a variety of rare genetic/genomic disorders. Particularly, chromosomal abnormalities/disorders have been considered in the rare disease context. Such approach to chromosomal abnormalities/disorders has been found to be rather new for an appreciable part of researchers and health care providers. To be more precise, a small number of rare chromosome diseases — presumably incurable genetic conditions — have been reported to be relatively successfully treated. Such a case has been already reported in the literature [45]. It is noteworthy that similar approaches to genetic disease therapy might be successful [46] in at least those cases of RTT, which are associated with CNV or mutations in genes other than *MECP2*.

Finally, numerous aspects of RTT diagnostic research were discussed. Diagnostic workflows used in different countries (including one used in Russia [47]) were presented. Here, it is important to mention that diagnostic success for patients suffering from RTT/*MECP2-*related diseases or any rare (monogenic) disease is highly dependent on the coverage of genetic and phenotype data within existing databases. Fortunately, a recent study has exactly confirmed this idea showing that online databases can be a promising starting point for researchers and health-care professionals who use these data [48].

## Conclusions

The attractiveness of the World Congresses on RTT evidences that RTT is a major focus of current biomedical research and represents a well-studied disease. The congress proceedings (see Additional file 1) further confirm this statement. Actually, there is a more or less clear view of how successful therapeutic interventions in this presumably incurable disease can be developed. This view is certainly the result of an extensive body of research. RTT studies have significantly contributed to rare disease research, as a whole. RTT research is consistently shown to be relevant to a broad spectrum of biomedical areas [10, 12, 18, 20, 22, 25, 40–43]. Taking a simple look at the number of articles dedicated to RTT in the most popular scientific databases (i.e. PubMed, Scopus, and Web of Science etc.) depicts that a body of RTT research is larger than an average research body dedicated to a genetic disease with a similar incidence. Undoubtedly, this is the result of exceptional and long-standing activity of RTT associations around the world. These associations have made a significant stimulation and contribution to RTT research, which has resulted to the aforementioned achievements. Thus, one can conclude the dissemination of RTT associations’ experience is important for international research and clinical community of rare disease specialists. The next (9th) World RTT Congress will be held on the Gold Coast in Queensland, Australia on the 30th of September until the 3rd of October 2020.

### Post scriptum

Sadly, we have to end our review by mentioning the untimely death of Professor Yuri B. Yurov during the preparation of this communication after courageous fight against a devastating disease. Yuri was at the forefront of organizing the VIII World RTT Congress & Symposium of Rare Diseases. He was probably the most recognized Russian researcher of RTT. Unfortunately, the review summarizing his many scientific accomplishments and describing his life unselfishly devoted to bioscience has not been included in the most popular scientific databases unlike many obituaries of other recognizable scientists, unhappily abundant this year. Accordingly, to redress this injustice, we have taken the opportunity to pay attention of a wider audience to the life and legacy of this prodigious researcher [49]. This tragedy is the major, but not unique, reason of the delayed publication of this article.

## Additional file

Additional file 1: Proceedings of VIII World Rett Syndrome Congress & Symposium of Rare Diseases. (PDF 821 kb)

## Abbreviations

CNV: Copy number variations
*MECP2*: Methyl CpG binding protein 2 gene
RTT: Rett syndrome
