# Supplementary material for: VIII World Rett Syndrome Congress & Symposium of rare diseases, Kazan, Russia
Source: Mol Cytogenet. 2018 Dec 24;11:61. doi: 10.1186/s13039-018-0412-2 (PMC6304760; doi:10.1186/s13039-018-0412-2)
Supplement: Supplementary file 1 — Proceedings of VIII World Rett Syndrome Congress & Symposium of Rare Diseases. (PDF 821 kb) [file 13039_2018_412_MOESM1_ESM.pdf]

Rett Syndrome
World Congress. May 13-17, 2016 | Kazan
Together above the dream

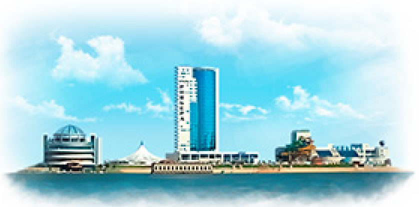

Congress Proceedings

VIII World Rett Syndrome Congress

&

Symposium of rare diseases

May 13-17, 2016, Kazan, Russia

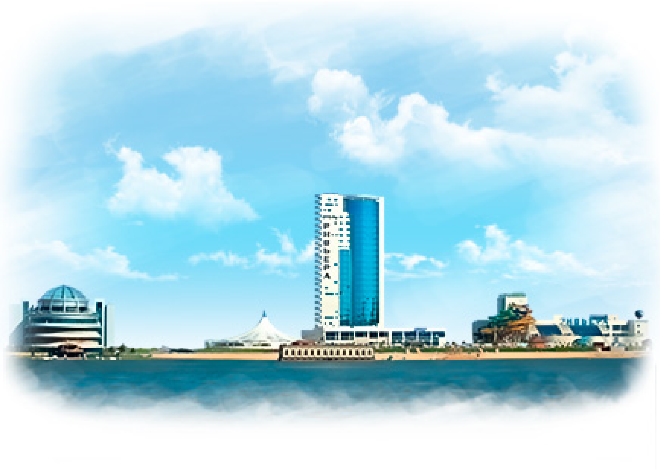

http://worldcongress2016.rettsyndrome.ru/

Rett Syndrome

World Congress. May 13-17, 2016 | Kazan

Together above the dream

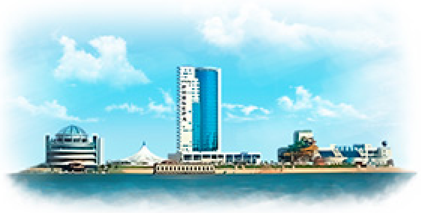

Editors:

Ivan Y Iourov (Moscow, Russia)

PhD, Doctor of Science, Professor of Medical Genetics, Head of Laboratory of Molecular Brain Genetics at Mental Health Research Center, Moscow

Svetlana G Vorsanova (Moscow, Russia)

PhD, Doctor of Sciences, Professor, Academician of Russian Academy of Natural History, Head of Laboratory of molecular cytogenetics of neuropsychiatric disorders at Research and Clinical Institute for Pediatrics at the Pirogov Russian National Research Medical University, Ministry of Health, Moscow

Yuri B Yurov (Moscow, Russia)

PhD, Doctor of Sciences, Professor, Academician of Russian Academy of Natural History, Head of Laboratory of cytogenetics and genomics of psychiatric disorders at Mental Health Research Center, Moscow

---

Technical editor (designed & drafted):

Maria A Zelenova (Mental Health Research Center, Research and Clinical Institute for Pediatrics named after Y.E. Veltishev at the Pirogov Russian National Research Medical University, Ministry of Health, Moscow, Russia)

Akcnowledgements: O.S. Kurinnaia (Mental Health Research Center, Research and Clinical Institute for Pediatrics named after Y.E. Veltishev at the Pirogov Russian National Research Medical University, Ministry of Health, Moscow, Russia)

How to cite:

1) **Authors of a proceedings paper** (Iourov IY, Vorsanova SG, Zelenova MA, Vasin KS, Kurinnaia OS, Shmitova NS, Ratnikov AM, Yurov YB.)
2) **Title of a proceedings paper** (Rare genomic disorders: a high-resolution whole-genome copy number variation (CNV) analysis of children with intellectual disability, congenital malformations and autism.)
3) **Authors/title of the congress paper** (In: Iourov IY, Vorsanova SG, Yurov YB, Bertrand T. VIII World Rett Syndrome Congress & Symposium of Rare Diseases, Kazan, Russia).
4) **Title of the journal:** Molecular Cytogenetics.
5) **Year:** 2018;
6) **Issue of Molecular Cytogenetics:** 11
7) **Number of the congress paper given when published:** X https://doi.org/10.1186/s13039-018-0412-2
8) **Number of proceedings' paper:** S54

Example:

Iourov IY, Vorsanova SG, Zelenova MA, Vasin KS, Kurinnaia OS, Shmitova NS, Ratnikov AM, Yurov YB. Rare genomic disorders: a high-resolution whole-genome copy number variation (CNV) analysis of children with intellectual disability, congenital malformations and autism. In: Iourov IY, Vorsanova SG, Yurov YB, Bertrand T. VIII World Rett Syndrome Congress & Symposium of Rare Diseases, Kazan, Russia. *Mol Cytogenet.* **2018**; *11*:X.S54.

1. Rett syndrome and *MECP2*

Autonomic Disturbances in Rett syndrome

Budden S.

Providence St Vincent's Hospital Portland Oregon USA.

Rett syndrome is a well-recognized neurodevelopmental disorder as a result of *MECP2* mutations. Research has clarified that there is impaired function in the grey matter involving cortical, cerebellar, and brain stem functions. In fact the supportive criteria for meeting the diagnosis of RTT includes autonomic disturbances such as breathing irregularities, air bloating and swallowing, bruxism, inappropriate laughing and screaming cold small purplish hands and feet, diminished response to pain and sleep disturbances. These signs and symptoms are of great concern to all families, care providers, school teachers and physicians who care for them. Hence a clearer understanding of these mechanisms will help them learn to manage individuals with Rett syndrome. This presentation will discuss the effects of *MECP2* mutations on cortical function, neuroanatomical, and biological mechanisms that result in autonomic dysfunction and tip the nervous system towards a Stress Response. Currently literature on Rett syndrome provides robust but isolated publications from independent work on neurotransmitter receptor abnormalities, effects on neuropeptides and synaptic disruption. The aim in presenting this paper is to understand the global changes that impact on

autonomic function in Rett syndrome and sets it apart as a challenge in management from other neuro-genetic disorders. Parents have specific concerns related to disturbances in sleep, respiratory system, cardiovascular system, gastrointestinal system and urinary tract dysfunction. It is imperative that those of us who care for these children have a clearer understanding on what to expect as they mature and how to manage their treatment daily at home, in school, in new surroundings with friends and people they do not know. This discussion will provide suggestions for management and provide a forum for open discussion with the audience.

References:

[1] McArthur AJ, Budden SS. (1998) Sleep dysfunction in Rett syndrome. A trial of exogenous Melatonin treatment. Dev Med Child Neurol 40. (3) 186-192

[2] Kerr AM, Julu PO. (1999) Recent insights into hyperventilation from the study of Rett syndrome. Arch Dis. Child 80:384-387

[3] Weese–Mayer DE, Lieske SP, Boothy CM, Kenny AS, Bennett HL, Ramirez JM. (2008) Autonomic dysregulation in young girls with Rett syndrome during nighttime in home- recordings 43: 1045-1060.

[4] Duncan JR, Patterson DS, Hoffman JM, Mokler DJ, Borenstein NS, Belliveau RA, Krous HF, Hass EA, Stanley C, Nattie EE, Trachtenberg FL, Kinney HC. (2010) Brain Stem Serotonergic Deficiency in Sudden Infant Death Syndrome. JAMA 303:5, 430-437.

A case of *MECP2* duplication syndrome in female, was found by MLPA genetic testing, with west syndrome and favoroble response of seizures on ACTG treatment.

Bulatnikova M.A.[1], Vasilishina A.A.[1], Kotelevskaya E.A.[1], Dvoeglazova M.O.[1], Kusnezova O.A.[2], Larionova V.I.[3]

[1]Pokrovskiy stem cell bank [2]Neurological department of clinic of Saint-peterburg pediatric medical University [3]North-west Medical University named after Mechnikov.

Lubs syndrome or mecp2 duplication syndrome is one of rare, but often recognosible genomic disorders, which is related with whole mecp2 duplication. Lubs syndrome is more frequent within males in contrast with rett syndrome, but a few cases of lubs syndrome in female were described. Clinical picture of mecp2 duplication include mental retardation with history of severe motor and speech delay during firth eyes of life and microcepaly, often autistic features, severe symptomatic epilepsy with onset with febrile seizures, recurent bronchitidis story and nosospecific craniofacial appearance. Clinical picture of lubs syndrome are not simular for rett syndrome releted with mecp2 microdeletion, motor and speech delay in cases of lubs syndrome begins with first mounths of life, there are not deceleration and behavior abnomalities are different.

A case of the girl with diagnosis of lubs syndrome, verifed by MLPA genetic testing with west syndrome and favoroble response of seizures on ACTG treatment are presented.

A two year old girl was directed to the consutation of geneticst from

neurological department with resistant to antiepileptic drugs epilepsy, microcephaly and global developmental delay, growth retardation, history of frequent pulmonary infections, and multiple congenital craniofacial abnomalities.

The pregnancy and delivery were normal. On the firth days of life diffuse muscle hypotonia and pure sucking were presented. Developmental delay was suscribed after tree month of life (pure holding of the head). Recurent bronchitis were presented almost each two months during firth year. Firth seizures were febrile, but to the nine month of life tranformation of seizures to infantile spasms without fever was been.

Neurologist, based on composition of infantile spasms and hypsarhitmia on EEG-monitoring and global developmental delay, established the diagnosis of symptomatic west syndrome. The treatment opf ACTG was initiated.

The girl was not sitting, rolling. There was pure eye fixation, speech was absent. Moderate growth and weight delay was detected. Craniofacial abnomalitiese include moderate microcephaly, high-arched thin eyebrows, almound-shaped eyes with short palpebral fissures, midface hypoplasia, hypoplasia of aleae nasi, high palate without clefting, thin upper lip, micrognatia, pointed chin. There were thin nails, and unspecific changing of palm creaces.

Taking in account developmental delay, craniofacial apperance and the history of recurent bronchitis, which were very simular on sings of atipical 22q11.2 deletion syndrome, 22q11.2 deletion were suspected. Genetic testing using MLPA kit P015 MRS-Holland was performed. No abnormal signal of 22q11.2 probes were detected,

but mecp2 duplication was found. Taking in account craniofacial signs, that we before we interpreteted firstly as 22q11.2 sings were descrebed as typical for lubs syndrome too, cronic pulmonary disorders are also one of sings of lubs syndrome and the most important for patient with mecp2 duplication is more common severe epelepsy course and cases with onset of epelepsy with febrile seizures was suscribed, the diagnosis of mecp2 duplication (lubs syndrome) was esteblished genotype-phenotype correlations analysis based on genotype-phenotype correlations analysis.

In conclution we want to take our attention on good response for ACTG treatment with elimination as seizures as hypsarrithmia during six month. After half of the year following by hypsarritmia elimination, she rolling and sitting independently, but speech was still absent. We are sure to collect the new data about clinical picture of lubs syndrome and following its systematization as for male as for female cases is neseassary for forming detaled recomendations for managment patients with this rare entity.

A Causative Link Between Hand Mannerism and Scoliosis in Rett Syndrome.

Lotan M.[1,2], Litbag A.[2], First U.[2]

[1]*National center for evaluation assessment and supervision for individuals with RS. Sheba hospital, Ramat-Gan, Israel* [2]*Physical Therapy Department, Ariel University, Ariel.*

Abstract. Scoliosis is the most common orthopedic co-morbidity in Rett syndrome. Overall, the literature is consistent with the fact that the majority of girls with Rett syndrome will develop scoliosis and occurrence prior to adolescence is not unusual [1]. One of the most affected sensory systems in Rett syndrome is the proprioceptive system [2]. The distorted sensory processing of incoming messages from this system imposes the person with RTT to base her movement abilities and postural control on miss-interpretation of signals coming from her own body. Experts in the field of RTT believe that such distorted messages leads to: Disorganized and misunderstood body scheme, apraxia, strange and unique (individual) postures in seating, standing and walking and scoliosis. Our knowledge of motor learning, as well as significant physical therapy approaches, and neurological rehabilitation principles suggest that in order for a body scheme to be constructed and properly performed, repetitive movements should be performed [3]. One of the more constant proprioceptive feedback for all individuals with RTT is their hand stereotypical movement performed during 30-80% of their waking hours [4]. It is therefore assumed that due to

ambiguous sensory feedback from the rest of the body, the person with RTT will tune-in to the clear continuous sensory messages sent to her brain by her hands, thereby gradually changing her postural alignment in-sink with the direction of the hands. The main assumption is that there is a connection between direction of hand mannerisms and scoliosis, and it is known that intrinsic feedback provided through sensory systems allow for motor adaptation.

Goal: To establish a connection between direction of Hand mannerisms and scoliosis

Procedure: 20 families with Rett syndrome were asked to deliver pictures of their daughters with 6 month interval. An evaluation of progression of hand mannerisms and development of scoliosis was performed. Results: A link was found between asymmetry of hand mannerisms presiding the direction of scoliosis by 2-10 years.

Conclusions: It seems that scoliosis in Rett syndrome is postural by nature, and is derived by fixation of an asymmetry pattern of hand mannerisms, later affecting the trunk.

Clinical implications: Early intervention which will involve changes in hand stereotypical movements, and preventing hand mannerisms from fixating on one constant pattern of hand movements, might influence (postpone or reduce the severity of, or even eliminate) the development of scoliosis. Future research programs are planned in order to evaluate this notion and implement appropriate intervention programs.

References:

[1] McClure MK, Battaglia C, McClure RJ. The relationship of cumulative motor asymmetries to scoliosis in Rett Syndrome. Am J Occup Ther. 1998;52:196-204.

[2] Lotan M. Tsury SP, Sinuani BZV. The proprioceptive system as the origin of fear of movement in Rett syndrome: Research and clinical implications. A seminar work, physical therapy department, Ariel University, Ariel, December 2-5, 2012.
[3] Raine S, Meadows l, Lynch-Ellerington M. Bobath Concept: Theory and Clinical Practice in Neurological Rehabilitation. Chichester: Wiley-Blackwell; 2009.
[4] Lotan M, Roth D. The Effect of Hand Vibrators on the Hand Stereotypes and Function in Rett Syndrome - Two Case Studies. Is Phys Quart. 1996;52:23-6. [Hebrew]

PROCEEDINGS S4

Concomitant duplication at 11p14.3 and deletion at Xq28 (*MECP2*) in a girl with atypical Rett syndrome

Kurinnaia O.S.[1,2,3], Vorsanova S.G.[1,2,3], Yurov Y.B.[1,2,3], Iourov I.Y.[1,2,4]

[1]Research and Clinical Institute for Pediatrics named after Y.E. Veltishev at the Pirogov Russian National Research Medical University, Ministry of Health, Moscow, Russian Federation [2]Mental Health Research Center, Moscow, Russian Federation [3]Moscow State University of Psychology and Education, Moscow, Russian Federation [4]Department of Medical Genetics, Russian Medical Academy of Postgraduate Education, Ministry of Health, Moscow, Russian Federation.

Using molecular karyotyping (NimblGen 135K platform) and a bioinformatic approach to definition of pathogenic values of genomic rearrangements described in details previously [1, 2], we have detected Xq28 (*MECP2*) deletion along with a duplication at 11p14.3 in a 6 year old girl with atypical Rett syndrome (RTT). The duplication involved *FANCF* associated with Fanconi anemia and involved in the Fanconi anemia pathway [KEGG ID: hsa03460]. To make genotype/phenotype correlations, we have retrospectively analyzed a cohort of 400 patients with speech and developmental delay, intellectual disability and/or autism. Among these patients, a duplication at 11p14.3 affecting

22 genes indexed in OMIM (online Mendelian inheritance in man) in a boy with developmental delay, microcephaly, short neck, low set ears, and widely set nipples was found. This duplication was larger than in a girl with RTT and was mosaic (affecting more than 25% of cells).

Xq28 (*MECP2*) deletion generally causes milder forms of RTT [1]. The index case appears to be more severe than those due to these deletions. We suggest that more severe phenotype results from concomitant duplication at 11p14.3. Since cases of concomitant chromosome abnormalities or genomic rearrangements are likely to be more common than previously recognized [2, 3], one can speculate that the index case is the result of simple coincidence. However, a kind of parental gonadal genome instability (described previously [3]) cannot be excluded. Nevertheless, RTT seems to be common enough to co-occur with another genetic disorder. A number of such cases have been already described in the literature [4]. *The study was supported by the Russian Science Foundation (project #14-35-00060).*

References:

[1] Iourov IY, Vorsanova SG, Voinova VY, Kurinnaia OS, Zelenova MA, Demidova IA, Yurov YB. Xq28 (MECP2) microdeletions are common in mutation-negative females with Rett syndrome and cause mild subtypes of the disease. Mol Cytogenet. 2013; 6(1):53.

[2] Vorsanova SG, Iurov IIu, Voinova VIu, Kurinnaia OS, Zelenova MA, Demidova IA, Ulas EV, Iurov IuB. [Subchromosomal microdeletion identified by molecular karyotyping using DNA microarrays (array CGH) in Rett syndrome girls negative for MECP2 gene mutations]. Zh Nevrol Psikhiatr Im S S Korsakova. 2013; 113(10):63-8.

[3] Vorsanova SG, Iourov IY, Demidova IA, Kirillova EA, Soloviev IV, Yurov YB. Chimerism and multiple numerical chromosome imbalances in a spontaneously aborted fetus. Tsitol Genet. 2006; 40(5):28-30.

[4] Vorsanova SG, Iourov IY, Yurov YB. Neurological, genetic and epigenetic features of Rett syndrome. J Pediatr Neurol. 2004; 2(4):179-190.

Development of Clinical Guidelines for the Management of Communication in Individuals with Rett Syndrome

Townend G.[1], Bartolotta T.[2], Garrett S.-A.[3], Urbanowicz A.[4], Wandin H.[5], Curfs L.[1]

[1]Rett Expertise Centre Netherlands - GKC, Maastricht University Medical Centre, Maastricht, The Netherlands [2]School of Health Sciences, Stockton University, Galloway, NJ, USA [3]Isle Talk, Killorglin, County Kerry, Republic of Ireland [4]University of Western Australia & Edith Cowan University, Perth, WA, Australia [5]Swedish Rett Centre, Frösön, Sweden.

Background: The number of specialist centres for the care & management of individuals with Rett syndrome (RTT) is growing internationally. There is huge variability in knowledge/expertise and clinical practice between and within countries [1, 2]. Guidelines are needed which provide information to families and which can be followed by clinicians.

Project and aim: In February 2016 an international consortium (see authors), led by the Rett Expertise Centre Netherlands, began a two-year project to develop international clinical guidelines for the management of communication (assessment, intervention and long-term management) in individuals with RTT. The project was made possible due to the financial support of a HeART Grant awarded by rettsyndrome.org.

Methodology: The project is based on well-established practices for the creation of evidence-based guidelines through a consensus approach. It follows the methodology used to develop the scoliosis [3], growth & nutrition [4], and bone health guidelines [5] for RTT. The project consists of five phases: ♣ Phases 1 & 2 (simultaneous): Review of

published and unpublished literature and gathering of available evidence ♣ Phase 3: Preparation of and consultation on draft guidelines ♣ Phase 4: Creation and dissemination of final guidelines ♣ Phase 5: Monitoring uptake and implementation of guidelines The involvement of family members of individuals with RTT and of professionals experienced in the field of communication and RTT is important to the shaping and effective implementation of the final guidelines. If you would like to know more about the project or wish to participate in the consultation process please contact Gill Townend - g.townend@maastrichtuniversity.nl

References:

[1] Townend, G.S., Smeets, E., van Waardenburg, D., van der Stel, R., van de Berg, M., van Kranen, H.J., & Curfs, L. M. (2015). Rett syndrome and the role of national parent associations within a European context. Rare Diseases and Orphan Drugs, 2(2), 17-26.

[2] Townend, G.S., van Kranen, H.J., van der Stel, R., van den Berg, M., Smeets, E., van Waardenburg, D., & Curfs, L.M. (2015). Rett Syndrome as a Rare Disease: A European Perspective. Public Health Genomics. 18(4), 233-236. doi: 10.1159/000381139.

[3] Downs, J., Bergman, A., Carter, P., Anderson, A., Palmer, G.M., Roye, D., at al. (2009) Guidelines for management of scoliosis in Rett syndrome patients based on expert consensus and clinical evidence. Spine (Phila Pa 1976), 34(17), E607-617. doi: 10.1097/BRS.0b013e3181a95ca4

[4] Leonard, H., Ravikumara, M., Baikie, G., Naseem, N., Ellaway, C., Percy, A., at al. (2013) Assessment and management of nutrition and growth in Rett syndrome. J Pediatr Gastroenterol Nutr, 57(4), 451-460. doi: 10.1097/MPG.0b013e31829e0b65

[5] Jefferson, A., Leonard, H., Siafarikas, A., Woodhead, H., Fyfe, S., Ward, L.M., et al. (2016) Clinical Guidelines for Management of Bone Health in Rett Syndrome Based on Expert Consensus and Available Evidence. PLoS One, 11(2), e0146824. doi: 10.1371/journal.pone.0146824

Don´t forget the gallbladder – Management of gallbladder disease in Rett syndrome

Freilinger M.[1], Anderson A.[2], Wong K.[2], Baikie G.[3,4], Ravikumara M. [5], Downs J. [2,6], Leonard H.[2]

[1]Department of Paediatrics and Adolescent Medicine, Medical University Vienna, Vienna, Austria [2]Telethon Institute for Child Health Research, Centre for Child Health Research, The University of Western Australia, Perth, WA [3]Department of Developmental Medicine, Royal Children's Hospital, Murdoch Childrens Research Institute, Melbourne, Vic. [4]Department of Paediatrics, University of Melbourne, Melbourne, Vic. [5]Department of Gastroenterology, Princess Margaret Hospital for Children, Perth, WA [6]School of Physiotherapy and Exercise Science, Curtin University, Perth, WA, Australia.

Rett syndrome (RTT) is a severe neurodevelopmental disorder caused by mutations in the X-linked methyl-CpG-binding-protein-2 (MECP2) gene with more and more well known clinical features. Although survival of females with Rett syndrome has improved since the late 1960s poor growth is still playing a centrally role affecting morbidity and mortality. Beside altered oropharyngeal dysfunction and problems with gastroesophageal motility such as reflux and constipation gallbladder disease was already reported early to be a problem in girls with RTT. The incidence of cholelithiasis and/or cholecystectomy in Rett Syndrome was 2.3 and 1.8 per 1000 person-years using the Australian Rett Syndrome Database and the International Rett Syndrome Phenotype Database respectively. The function of the gallbladder was assessed in a clinical cohort and revealed a mean contractility index smaller than for healthy individuals but similar to children with Down syndrome. Gallbladder disease is relatively common in

RTT and should be considered in the differential diagnosis of abdominal pain after excluding gastroesophageal reflux. This and other recommendations for assessment and management of gallbladder disease were identified with the Delphi technique.

Emotion and Behavior in Rett syndrome

Budden S.

Providence St Vincent's Hospital Portland Oregon USA

MECP-2 mutations in Rett syndrome (RTT) have a distinct effect on brain maturation causing cortical and autonomic disturbance reflected in changing emotion and behavior. Infants appear quiet, sleepy, with a poor suck and cry. Later loss of language followed by loss of hand function and onset of stereotypes occurs. Frequently intermittent eye crossing, anxiety, irritability, agitation and screaming with hair pulling, biting or hitting occur with associated hyperactivity, rapid random pacing, and toe walking. Sleep disturbances associated with short periods of laughing or screaming and breathing problems become obvious. Adolescent girls demonstrate increasing gaze intensity, loud moaning and screaming. Later; moodiness, sleeplessness, poor appetite, loss of weight, lack of interest and unexplained crying may occur, suggesting possible depression. The biological underpinnings of emotion and behaviour result from dysfunctions in mono-aminergic systems and are age related .Neuropathological studies show high binding of Serotonin

type I and II receptors in the brain stem reflecting the immaturity of the neurons. Neurochemical changes alter synaptic function. Hypo-function of monoamines, are present as early as 36 weeks of gestational resulting in early placidity. Agitation may result from increased levels of glutamate levels and Cortisol. Corticotrophin release factor-CRF- is documented in RTT mouse model. Neurotransmitter abnormalities such as elevated B- endorphins and decreasing levels of biogenic amines occur with age. Impaired autonomic nervous system result in respiratory disturbances, agitation, panic-like attacks, disordered arousal and sleep, mood changes, intermittent strabismus, tremors, myoclonic jerks, abnormal motor activity, gastrointestinal dysfunction, vasomotor changes, cardiac irregularities and fluctuating blood pressure. Undesired behaviours may result from unrecognized conditions such as seizures, dental problems, ear infections, reflux, constipation, gall stones, renal stones, fractures, dystonic spasms at night, menstrual discomfort, sleep apnoea and day time sleepiness. In maturing girls depression is suspected based on history of sleeplessness, poor appetite, weight loss, lack of interest in activities they previously enjoyed. Other reasons for unexplained crying, sadness, loneliness can result from changes in school, in care givers, loss of social contacts and school peers. Sometimes agitation and negative reaction result from changes in daily routine and unrecognized abuse. Clinicians must remain alert to making a correct diagnosis. Individuals using augmentative communication systems can communicate their feelings and emotion. A knowledgeable team

can assist in addressing these issues in the most effective way and make a major difference in the person's life.

References:

[1] Samaco, R. C.; Mandel-Brehm, C.; Chao, H; et al. Neul, J. L. (2009). "Loss of MeCP2 in aminergic neurons causes cell-autonomous defects in neurotransmitter synthesis and specific behavioral abnormalities".

[2] Budden SS, Myer EC, Butler IJ.(1990) Cerebrospinal fluid studies in Rett syndrome: biogenic amines and beta-endorphins. 12(1);81-84.

[3] Zoghbi H, Percy AK, Glaze DG, Butler IJ, Riccardi VM.(1985) Reduction in Biogenic Amine Levels in Rett syndrome. N Engl J Med 313:921-924.

[4] McGill BE, Bundle SF, Yaylaoglu M B., Carson JP, Thaller C, Zoghbi H. Y. (2006). Enhanced anxiety and stress-induced corticosterone release are associated with increased Crh expression in a mouse model of Rett syndrome. Proc. Natl. Acad. Sci.103: 18267–18272.

Epilepsy in patients with typical variant of Rett syndrome and MeCP2 mutations: results of own observation of 11 cases

Bobylova M.Yu.[1], Ivanova I.V.[2], Nekrasova I.V.[2], Mukhin K.Yu.[1], Pylaeva O.A.[1], Kholin A.A.[3], Iljina Ek.S.[3], Kulikov A.V.[4], Iljina El.S.[4], Nesterovsky Yu.E.[3]

[1]Svt. Luka's Institute of Child Neurology and Epilepsy, Moscow [2]Russian Children's Clinical Hospital, Ministry of Health of Russia, Moscow [3]Department of Neurology, Neurosurgery, and Medical Genetics, Pediatric Faculty, N.I.Pirogov Russian National Research Medical University, Ministry of Health of Russia, [4]The Mental Health Research Center of the Russian Academy of Medical Science, Moscow Objective.

Studying data of anamnesis, clinical state, electro-encephalographic, brain MRI in patients with Rett syndrome (MECP2). Material and methods. We studied 11 patients (female) from three to 23 years old with Rett syndrome and MeCP2 mutations. Observation continued 10 years (2006-2015). We analyzed the results of the neurological status, night sleep

video-EEG monitoring, MRI. Results and conclusions. Epilepsy diagnosed in six cases (54, 5%). The overage age of debut of epileptic seizures was 3 years 9 months. There are some types of seizures: generalized, myoclonic, myotonic, tonic, versive, focal motor, atypical absences. Status epilepticus evolved in one patient. Generalized seizures were 56, 25%, focal seizures – 43, 75%. EEG changing marked in nine patients (81, 8%): slowdown back activity, episodes of periodic regional slowdown, regional epileptiform activity, and diffuse epileptiform activity like benign focal epileptiform discharges (BFED). five patients took antiepileptic drugs. All of them had improved during treatment. There were reducing of frequency of the seizures up 50% - 4 cases (80%). one patients with resistant epilepsy was taken combination of drugs (levetirecetam, topiromat, zonisamide, benzodiazepine) with stopping of seizures in the night sleep and decreasing of frequency of daytime seizures to 50%. We believe there is very important of study epilepsy in patients with Rett syndrome and improvement of its treatment. Conclusion. In our study changes of EEG were detected in the majority of patients with Rett syndrome. Regional and diffuse epileptiform activity during wakefulness was predominantly localized in the central parts, with a tendency to an increase in the representation during sleep. Epileptic seizures were observed in about half of patients, it was noted as a generalized and focal polymorphic attacks. In all cases shown AET efficiency. It was reached remission (complete absence of seizures during follow-up) in 50% of Rett syndrome patients with epilepsy. 50% of patients achieved an improvement in the form of a

reduction in seizure frequency of 50% or more. Unfortunately it must be noted that at the present time in Russia there is no specialized state clinic or department specifical for patients with Rett syndrome. Data about epilepsy and its treatment during scattered and not well known. Features of the EEG and the clinical picture of epilepsy in Rett syndrome requires further study. Rett syndrome is a systemic disease. Therefore, a multidisciplinary approach is important, as well as the cooperation of doctors of different specialties.

Key words: Rett syndrome, MECP2, epilepsy, video-EEG monitoring

PROCEEDINGS S9

Functioning in children with Rett syndrome and idiopathic autism: a comparative study

Zelenova M.A.[1,2,3], Kazmin A.M.[3], Vorsanova S.G.[1,2,3], Yurov Y.B.[1,2,3], Iourov I.Y.[1,2,4]

[1]Mental Health Research Center, Moscow, Russian Federation [2]Research and Clinical Institute for Pediatrics named after Y.E. Veltishev at the Pirogov Russian National Research Medical University, Ministry of Health, Moscow, Russian Federation [3]Moscow State University of Psychology and Education, Moscow, Russian Federation [4]Department of Medical Genetics, Russian Medical Academy of Postgraduate Education, Ministry of Health, Moscow, Russian Federation.

Psychological studies of Rett syndrome (RTT) remain an important part of RTT research and healthcare [1]. Similarly, idiopathic autism ought to involve these studies along with genetic testing to reveal possible etiological factors and pathogenic mechanisms of the disease, and develop therapeutic (intervention) strategies. In present study, we assessed a

cohort of 18 children aged from 1 year 6 months to 13 years consisting of 10 girls with RTT (all the girls have *MECP2* mutations) and 8 children with «classical» autism without detectable genetic defects. Firstly, we performed psychological evaluation using psychoeducational profile (PEP) [2]. Furthermore, according to a mental age of each individual, we found psychological age-matching children with idiopathic autism to perform a comparative study. The evaluation was completed by using a modified "Functionality 07 (F07)" questionnaire, which is an assessment scale based on ICF-CY (International Classification of Functioning, Disability and Health) [3]. Statistical analysis was performed using Mann-Whitney test (Statistica 8 software). Individuals with RTT scored better considering “involvement in sensory research” ($p<0.05$) and “engagement” ($p<0.05$) criteria but were less successful at “play activity” ($p<0.01$) than children with idiopathic autism. These results allow us to conclude that children with RTT possess both developmental resources and substantial limitations.

We speculate that the latter may be a consequence of global changes to genome activity (transcriptional repression) due to *MECP2* mutations. Here, we have demonstrated the potential of psychological examination, which allows evaluating as disease severity as different aspects of the child’s functioning for defining psychological consequences of molecular genetic pathology. Apparently, the most promising direction in this field would be to use a united approach to the research, combining data from different fields, such as the one presented in ICF-CY. Consequently, these data would be

useful for determination of molecular and cellular basis for behavioral phenotypes in a variety of brain diseases as suggested previously [4, 5]. *The study was supported by the Russian Science Foundation (project #14-35-00060).*

References:

[1] Killian JT Jr, Lane JB, Lee HS, Pelham JH, Skinner SA, Kaufmann WE, Glaze DG, Neul JL, Percy AK. Caretaker quality of life in Rett syndrome: disorder features and psychological predictors. Pediatr Neurol. 2016. pii: S0887-8994(15)30314-3.

[2] Schopler E, Reichler RJ. Psychoeducational Profile. 1976.

[3] World Health Organization. International classification of functioning, disability and health. Geneva, WHO. 2001.

[4] Iourov IY, Vorsanova SG, Yurov YB. Chromosomal variation in mammalian neuronal cells: known facts and attractive hypotheses. Int Rev Cytol. 2006; 249:143-91.

[5] Vorsanova SG, Iurov IIu, Demidova IA, Voinova-Ulas VIu, Kravets VS, Solov'ev IV, Gorbachevskaia NL, Iurov IuB. [Variations of heterochromatic chromosomal regions and chromosome abnormalities in children with autism: identification of genetic markers in autistic spectrum disorders]. Zh Nevrol Psikhiatr Im S S Korsakova. 2006; 106(6):52-7.

Gene therapy using a self-complementary AAV9 (scAAV9) construct expressing a codon-optimized Mecp2 transgene in Mecp2-deficient mice improves survival, behavioral deficits and rescues breathing defects.

Matagne V., Villard L., Roux J.C.

UMR_S910, Medical Genetics and Functional Genomics, Marseille

Rett syndrome (RTT) is an X-linked neurodevelopmental disorder primarily affecting CNS functions. Most RTT cases are due to mutations in the methyl CpG binding protein 2 (*MECP2*) gene, a

global transcriptional modulator. There is currently no cure for the disease and drugs alleviating symptoms are the only available therapies. Recently, two different publications reported that gene therapy using Mecp2-deficient mice partially cured the disease (Gadalla et al 2013; Garg et al 2013). Although both studies showed some rescuing effect, the benefits seemed to be very variable. In order to try and improve transgene expression, we designed a plasmid construct expressing a codon-optimized version of Mecp2 that was used to generate a scAAV9 therapeutic vector. Thirty day-old Mecp2-deficient (KO) male mice were injected with the vector through the tail vein (2X10E11 vg/mouse). The treatment significantly increased survival (median survival 58d vs 99d in Mecp2 KO vs treated) and some behavioral deficits (Open field, Rotarod). Apneas, which are part of the characteristic RTT breathing defects, were also significantly reduced (125±51 vs 6±3, Mecp2 KO vs treated). This improvement could be due to the increased percentage of Mecp2-expressing cells that we found in the posterior brain areas involved in breathing regulation such as the locus coeruleus (23.8±4.7%) compared to more anterior ones (10.9± 1.4% in the cortex). These data indicate that even a low level Mecp2 expression can improve RTT symptoms in Mecp2-deficient mice. Further studies will aim at investigating the therapeutic effect of the same viral construct in female RTT mice.

HDAC6 Inhibitors, a novel therapeutic approach for Rett syndrome

Sangani N.B.[1], Gold W.A.[1,2], Lacina T.[3], Williamson S.[1], Cantrill L.C.[2,4], Kozikowski P.A.[5], Vallerini G.P.[5], Christodoulou J.[6,7]

[1]Genetic Metabolic Disorders Research Unit, Western Sydney Genetics Program, Children's Hospital at Westmead, Sydney, Australia [2]Discipline of Paediatrics & Child Health, The University of Sydney, Australia [3]Hochschule Mannheim - University of Applied Sciences, Faculty of Biotechnology, Mannheim, Germany [4]Microscope Facility, Kids Research Institute, Children's Hospital at Westmead, Sydney, Australia [5]Drug Discovery Program, Department of Medicinal Chemistry and Pharmacognosy, University of Illinois, Chicago, United States. [6]Department of Paediatrics, University of Melbourne, Australia [7]Neurodevelopmental Genomics Research Group, Murdoch Children's Research Institute, The Royal Children's Hospital, Melbourne, Australia.

Microtubule dysfunction, as one of the common endpoints in neurological disorders including Rett syndrome, results in impairment of microtubule transport, potentially triggering catastrophic defects in synaptic plasticity. In neuronal cells the microtubule network is essential for delivery of key molecules including BDNF, neurotransmitters and mitochondria to the synapse, as well as providing an architecture to support axonal and dendritic stability and dynamics. It has been demonstrated that the establishment and maintenance of the microtubule network is tightly controlled by post-translational modifications, most notably acetylation, which has a pivotal role. MeCP2 deficiency is associated with reduced levels of tubulin acetylation and the overexpression of histone deacetylase 6 (*HDAC6*), an enzyme that removes acetyl groups from α-tubulin, resulting in reduced microtubule stability. This finding

highlights the putative role of HDAC6 inhibitors as an emerging therapeutic strategy for Rett syndrome. Recent in vitro approaches using HDAC6 inhibitors demonstrate the improvement of microtubule–based transport processes in neuronal networks. Our current research approach involves investigating the therapeutic effects of a novel HDAC6 inhibitor in a Rett syndrome mouse model. We demonstrate that after two weeks of daily injections, both pre-symptomatic and symptomatic Mecp2T158A hemizygous male mice, showed improvement in their motor and coordination abilities, measured by open field and rotarod tests. In addition, we also assessed their respiratory function using whole body plethysmography and observed a dramatic reduction in the number of apnoeic episodes relative to the placebo-treated mice. In conclusion, our findings reveal that HDAC6 inhibition can improve motor, behaviour and respiratory deficits in a Rett syndrome mouse model and thus provide a promising therapeutic option for Rett syndrome patients.

PROCEEDINGS S12

Home-based, physical therapy, telehealth consultation in individuals with Rett syndrome – a pilot study

Lotan M.[1,2], Elefant C.[3], Downs J.[4,5]

[1]National center for evaluation assessment and supervision for individuals with RS. Sheba hospital, Ramat-Gan, Israel [2]Physical Therapy Department, Ariel University, Israel. Ariel [3]Head of Graduate school for Creative Arts Therapies, University of Haifa, Israel [4]Telethon Kids Institute, The University of Western Australia [5]School of Physiotherapy and Exercise Science, Curtin University.

Introduction. Rett syndrome (RTT) is a developmental disorder, affecting mainly females, caused by MECP2 mutations usually resulting in severe disability [1]. Due to the challenges faced by the individual with RTT and her family, her rehabilitation program should support her throughout different daily activities, contexts, and surroundings. Nevertheless, in the vast majority of cases, hands-on therapeutic intervention opportunities are available for the client during a minute part of her waking hours. Hence, a supplementary system is required in order to engulf the child with a comprehensive network of support. In some cases, the therapeutic interventions suggested for the person with RTT are minimal or non-existent. In such cases the primary caregivers should implement a supplementary/home-based intervention that will enhance the client's ability (or at least preserve the present state until a more intensive adapted therapeutic program is initiated) [2]. This pilot study suggests delivering physiotherapy support for RTT over long distances, which is a promising intervention mode suggesting promising results [3,4].

Methods. Five families of individuals with Rett syndrome living in Ireland participated in a six month bi-monthly home-based program via Skype consultation sessions by the first author residing in Israel who supported the program. Program results were evaluated through the use of Goal Attainment Scale (GAS) and followed a Participatory Action Research (PAR) model, a collaborative study design where all participants take part in

assessing, goal setting, acting, reflecting and evaluating progress.

Results. All participants showed improvements in achieving their set goals (according to GAS), parent and caregivers were extremely satisfied with the engagement over large geographical distances.

Conclusions. This presentation will discuss a model for physical therapy telehealth consultations in home-based intervention, which was applied by parents/caregivers, of children with RTT in accordance to the child's abilities and the family's possibilities. The presented intervention took place in Ireland and was supervised over Skype meetings from Israel. All participant (presenting different degrees of severity) showed significant improvements thereby, supporting authors' belief that constructing a home-based intervention program is essential for the person with RTT to achieve her best state of being. The GAS was found a useful tool to evaluate clients' progress. Participatory Action Research (PAR) was found as a useful model when implementing such a program.

References:

[1] Fehr S, Bebbington A, Nassar N, et al. Trends in the Diagnosis of Rett Syndrome in Australia. Ped Res. 2011;70:313-9.

[2] Rickards AL, Walstab JE, Wright-Rossi RA, Simpson J, Reddihough DS. A randomized, controlled trial of a home-based intervention program for children with autism and developmental delay. J Dev Behav Ped. 2007;28:308-16.

[3] Di Cerbo A, Morales-Medina JC, Palmieri B, Iannitti T. Narrative review of telemedicine consultation in medical practice. Patient preference and adherence 2015;9:65-75.

[4] Hailey D, Roine R, Ohinmaa A, Dennett L. The status of telerehabilitation in neurological applications. Jf telemed telecare. 2013;19:307-10

Losing and regaining independent walking in individuals with Rett syndrome

Lotan M.[1,2], Shtraus T.[1], Venetzian M.[1]

[1]Physical therapy department, Ariel University, Ariel, Israel [2]National center for evaluation assessment and supervision for individuals with RS. Sheba hospital, Ramat-Gan, Israel.

Background: Rett syndrome (RTT) is a genetic disorder affecting mainly women. The syndrome is characterized by four stages: I-Onset, II- Rapid Destructive Stage III- Plateau Stage/pseudo stationary, and IV-Late motor deterioration [1]. The clinical knowledge suggests that many with RTT capable of independent walking are experiencing episodes of losing and regaining independent walking, yet this phenomenon is scarcely mentioned in the literature [2]. Therefore, therapist treating this population might assume a child has reached the IV's stage of RTT, and refrain from trying to re-achieve walking.

Objectives: To evaluate the incidence of losing and regaining independent walking in individuals with RTT.

Procedure: An interview conducted with 49 parents of individuals with RTT showing independent walking.

Results: Eighty percent (36 of 49) have experienced losing and regaining independent walking at a mean age of 11.2 years. The causes attributing to losing independent walking have been found as: orthopedic (24%), neurological (17%), illness/medical (15%), sensorial (15%), emotional (11%), iatrogenic (9%), miscellanies (3%). Regaining walking was

achieved (after a mean of 193 days) due to intense intervention initiated by either parents or physiotherapists. **Conclusions:** The findings suggest that losing and regaining independent walking is a common phenomenon in RTT. Lose of functional abilities at adolescence is common among other individuals with special needs (Down syndrome, Cerebral Palsy) [3]. Moreover, no known anatomical or physiological attributes have been associated with the decline in motor abilities associated with the IV's stage of RTT. Therefore, the researchers suggest that the fourth stage (IV) described as an integral part of RTT should be discarded, and the three stages describing the development of RTT, should be referred to as suggested by Dr. Alison Kerr: Pre-regression, regression, and post regression [4].

References

[1] Ellaway C, Christodoulou J. Rett Syndrome: clinical Characteristics and Recent Genetic Advances. Disabil Rehabil.2001;23:98-106.

[2] Lotan M, Gutman A. Regaining walking ability in individuals with RS – A case study. Int J Child Health Hum Dev. 2012;11(2):163-9.

[3] Opheim A, Jahnsen R, Olsson E, Stanghelle JK. Walking function, pain, and fatigue in adults with cerebral palsy:a 7-year follow-up study. Dev Med Child Neuro. 2009;51:381–8. 4. Kerr AM. A Critical Account of Clinical and Physiological Studies in Rett Syndrome. University of Edinburgh. 2005.

PROCEEDINGS S14

MECP2 Duplication: genetic and clinical study in Spanish patients

Blasco Pérez L.[1], Vidal Falcó S.[1], Gean Molins E.[2], del Campo Casanelles M.[3], Català Cahís V.[4], Guillén E.[5], Lapunzina P.[6], Lloveras E.[7], Maortua H.[8], Martinez Castellano F.[9], Mesas

Aróstegui A.[10], Nevado J.[6], Obón Ferrer M.[11], Orellana Alonso C.[9], Plaja A.[12], Roselló Piera M.[9], Tejada M.I.[8], Tizzano E.[13], Armstrong Morón J.[14]

[1]Fundación San Juan de Dios, Barcelona [2]Unidad de consejo genético, Hospital Universitario San Juan de Dios, Barcelona [3]Pediatrics, Genetic Epidemiology, Hospital Valle Hebrón, Barcelona [4]Unitad de genética médica i biologia celular, Universidad Autonoma de Barcelona, Barcelona [5]Unidad de Genética, Hospital Virgen de la Arrixaca, Murcia [6]Instituto de Genética Médica y Molecular, Instituto de Investigación Sanitaria del Hospital Universitario La Paz, Madrid [7]Departamento de genètica, LABCO-Iberia [8]Laboratorio de Genética Molecular, Servicio de Genética, Hospital Universitario de Cruces, Bilbao [9]Unidad de genètica, Hospital Universitario y Politécnico La Fe, Valencia [10]Gastroenterologia, Hospital Xanit, Málaga [11]Area de Genètica clínica i Consell Genètic, Laboratoris ICS, Girona [12]Institut de Recerca (VHIR), Universitat Autònoma de Barcelona, Hospital Universitari Vall d'Hebron, Barcelona [13]Area Genetica Clínica y Molecular, Hospital Universitari Vall d'Hebron, Barcelona [14]Servicio de Genética Bioquímica&Rett, Hospital Universitario San Juan de Dios, Barcelona.

Objective: The MECP2 duplication syndrome (OMIM 300260) is a neurodevelopmental disorder X- linked characterized by severe to profound intellectual disability, early infantile hypotonia, autistic traits, seizures and recurrent infections. It usually affects boys, but also there are girls affected. The duplication origin could have a maternal or de novo inheritance. It has been reported about 120 cases all over the world, without a known incidence.

Material and Method: The aim of the study is to characterize a Spanish cohort with MECP2 duplication syndrome to improve our knowledge of the disease and perform a genotype-phenotype correlation.

Results: The cohort consists in 15 patients of both sexes diagnosed in different Spanish hospitals. The duplications were detected by MLPA and / or CGH-array. Our workflow was: 1) Check the duplication by qPCR-doses, and study XCI; 2) If FISH confirms tandem duplication, we detect the

breakpoints through PCR-long and Sanger sequencing; 3) Study the expression of the two MeCP2 isoforms by RT-qPCR; 4) Correlate the clinical presentation with the MECP2 duplicated region.

Conclusions: In this collaborative study has been characterized a heterogeneous cohort formed by patients of both sexes, with different phenotypes (Rett-like and duplication patients) and different inheritance of the mutation (maternal or de novo). This variety has allowed us to provide the necessary information to create a genotype-phenotype correlation. We suppose MECP2 duplication syndrome is an underdiagnosed disease that needs further characterization studies in order to give a better genetic and clinical diagnosis.

References:

[1] Van Esch, H. (2008, Update 2014). MECP2 Duplication Syndrome. GeneReviews®.

[2] Ramocki, M.B., Tavyev,Y.J., Peters, S.U. (2010). The MECP2 duplication syndrome. Am. J. Hum. Genet. 152A:1079-1088.

PROCEEDINGS S15

MECP2 interactome

Iourov I.Y.[1,2,3], Novikov A.Y.[1]

[1]Mental Health Research Center, Moscow, Russian Federation [2]Research and Clinical Institute for Pediatrics named after Y.E. Veltishev at Pirogov Russian National Research Medical University, Ministry of Health, Moscow, Russian Federation [3]Department of Medical Genetics, Russian Medical Academy of Postgraduate Education, Ministry of Health, Moscow, Russian Federation.

Understanding molecular and cellular roles of *MECP2* is crucial for uncovering intrinsic mechanisms and developing therapies of Rett syndrome (RTT). Despite significant success in *MECP2* biology, there are still numerous gaps in our understanding of its molecular and cellular functions as well as

functional consequences of *MECP2* mutations and/or copy number changes [1]. For instance, interactome and pathways analyses are exclusive in current biomedical literature, even though these bioinformatics methodologies have been shown to be rather informative for uncovering molecular and cellular functions and disease pathways [2-4]. To fill this gap in *MECP2* biology, we have performed an extensive analysis of *MECP2* interactome.

Using a previously described methodology of *in silico* molecular cytogenetics [3] and interactomic analysis [4], *MECP2* was addressed. According to BioGPS (http://biogps.org/), *MECP2* is highly expressed in whole human brain with higher expression values in the cerebellum and retina. This allows speculations that processes involving *MECP2* transcripts are likely to be brain-specific, especially in cases of defective *MECP2* functioning. *MECP2* interactome includes 43 main elements (genes/proteins), which are associated with 702 GO (gene ontology) entities/classes. Classifying GO entities according to their involvement in the *MECP2* interactome (i.e. pathways involving an element of the interactome more than 3 times), 84 GO entities were selected as those having relevance to pathways linked to *MECP2* interactome. Among these GO entities, we found 12 pathways, which were not previously attributed directly to *MECP2*: protein binding, RNA binding, zinc ion binding, ATP binding, enzyme binding, small molecule metabolic process, DNA repair, metal ion binding, Notch signaling pathway, cellular response to DNA damage stimulus, mitotic cell cycle, double-strand break repair via homologous recombination.

Interestingly, these pathways can be considered an appropriate target for molecular/cellular therapeutic interventions.

Recently, similar bioinformatic technologies were relatively successful not only for a description of genetic disease mechanism, but also for suggesting a therapeutic strategy. The latter was found applicable for treating cases of presumably incurable genetic conditions [5]. Taking into account the power of these systems biology (medicine) approaches to uncover disease mechanisms [2-6], we found pertinent to speculate about similar possibilities in RTT. Correcting molecular and cellular processes altered by a genetic defect (i.e. *MECP2* mutations or copy number variations) as suggested previously [5, 6], one can succeed in phenotypic recovery in RTT girls.

The study was supported by the Russian Science Foundation (project #14-15-00411).

References:

[1] Lombardi LM, Baker SA, Zoghbi HY. MECP2 disorders: from the clinic to mice and back. J Clin Invest. 2015; 125(8):2914-23.

[2] Sevimoglu T, Arga KY. The role of protein interaction networks in systems biomedicine. Comput Struct Biotechnol J. 2014; 11(18):22-7.

[3] Iourov IY, Vorsanova SG, Yurov YB. *In silico* molecular cytogenetics: a bioinformatic approach to prioritization of candidate genes and copy number variations for basic and clinical genome research. Mol Cytogenet. 2014; 7(1):98.

[4] Menche J, Sharma A, Kitsak M, Ghiassian SD, Vidal M, Loscalzo J, Barabási AL. Disease networks. Uncovering disease-disease relationships through the incomplete interactome. Science. 2015; 347(6224):1257601.

[5] Iourov IY, Vorsanova SG, Voinova VY, Yurov YB. 3p22.1p21.31 microdeletion identifies CCK as Asperger syndrome candidate gene and shows the way for therapeutic strategies in chromosome imbalances. Mol Cytogenet. 2015; 8:82

[6] Iourov IY, Vorsanova SG, Yurov YB. Single cell genomics of the brain: focus on neuronal diversity and neuropsychiatric diseases. Curr Genomics. 2012; 13(6):477-88.

MECP2 mutations cause atypical X chromosome replication and bi-allelic expression of Xq28 genes in Rett syndrome females

Yurov Y.B.[1,2,3], Vorsanova S.G.[1,2,3], Kolotii A.D.[1,2], Soloviev I.V.[1], Iourov I.Y.[1,2,4]

[1]Mental Health Research Center, Moscow, Russian Federation [2]Research and Clinical Institute for Pediatrics named after Y.E. Veltishev at the Pirogov Russian National Research Medical University, Ministry of Health, Moscow, Russian Federation [3]Moscow State University of Psychology and Education, Moscow, Russian Federation [4]Department of Medical Genetics, Russian Medical Academy of Postgraduate Education, Ministry of Health, Moscow, Russian Federation.

Alterations in X chromosome replication has been previously noted in Rett syndrome (RTT) [1-3]. However, RTT association with *MECP2* mutations has changed the research focus to *MECP2* biology leaving aside studying epigenetic alterations to chromosome X specific for this disease. Still, data on dysregulation of replication and transcription of inactive chromosome X in RTT in the light of *MECP2* involvement in transcriptional regulation [3] suggest that there should be a link between *MECP2* mutations and these epigenetic changes.

Here, we have correlated data on X chromosome replication timing assessed by interphase fluorescence *in situ* hybridization (FISH) [3-6] and data on *MECP2* mutations (R306C and R306X) and X chromosome inactivation patterns. Xq23 and Xq28 on the inactive chromosome X were shown to contain early replicating or transcriptionally active loci in RTT. Using interphase FISH with *in silico* topological analysis of signal distribution within nuclear

area [5,6], it has been demonstrated that DNA probes for Xq28 (anonymous clone PAC 24.23.0 and PAC 671D9, containing *MECP2*) shows synchronous patterns of DNA replication in a larger proportion of cells in RTT girls with R306C and R306X mutations in *MECP2*. Thus, these chromosomal locus exhibits late replication, which probably cause X chromosome inactivation escaping in at least a small population of blood cells in RTT patients. Similar replication patterns were not ever observed in individuals without *MECP2* mutations. In all the cases, X chromosome inactivation patterns were shown to be random (non-skewed). Therefore, it is likely that epigenetic alterations detected in these cases are locus-specific. Alterations to DNA replication have been previously shown to be involved in pathogenic cascades of brain diseases [6, 7]. However, there is not a positive association of late replication, and X chromosome inactivation escaping with a specific genetic defect. Therefore, *MECP2* mutations can be considered the first proven genetic causes of atypical X chromosome replication and bi-allelic expression of Xq28 genes in RTT. Since these epigenetic phenomena can be affected by exogenous interventions, these data appear intriguing in the light of possible molecular therapies in RTT. This study was supported by the Russian Science Foundation (project #14-15-00411).

References:

[1] Kormann-Bortolotto MH, Webb T. Alterations in replication timing of X-chromosome bands in Rett syndrome. J Intellect Disabil Res. 1995; 39(Pt 2):91-6.

[2] Vorsanova SG, Demidova IA, Ulas VYu, Soloviev IV, Kazantzeva LZ, Yurov YuB. Cytogenetic and molecular-cytogenetic investigation of Rett syndrome:

analysis of 31 cases. Neuroreport. 1996; 8(1):187-9.
[3] Vorsanova SG, Yurov YB, Kolotii AD, Soloviev IV. FISH analysis of replication and transcription of chromosome X loci: new approach for genetic analysis of Rett syndrome. Brain Dev. 2001; 23 Suppl 1:S191-5.
[4] Iourov IY, Vorsanova SG, Yurov YB. Fluorescence intensity profiles of in situ hybridization signals depict genome architecture within human interphase nuclei. Tsitol Genet. 2008; 42(5):3-8.
[5] Vorsanova SG, Yurov YB, Iourov IY. Human interphase chromosomes: a review of available molecular cytogenetic technologies. Mol Cytogenet. 2010; 3:1.
[6] Yurov YB, Vorsanova SG, Iourov IY. The DNA replication stress hypothesis of Alzheimer's disease. ScientificWorldJournal. 2011; 11:2602-12.
[7] Tiganov AS, Iurov IuB, Vorsanova SG, Iurov IIu. [Genomic instability in the brain: etiology, pathogenesis and new biological markers of psychiatric disorders]. Vestn Ross Akad Med Nauk. 2012;(9):45-53

Mitochondrial dysfunction, impaired antioxidant enzymatic activity and compromised proteasome function are involved in RTT cells oxidative damage

Valacchi G.

University of Ferrara

Introduction: In the last few years, a strong relationship between oxidative stress (OS) and Rett syndrome (RTT), a rare and orphan progressive neurodevelopment disorder affecting girls almost exclusively, has been well documented. However, to date the source of OS and the effects of the redox

imbalance in this disorder remain to be explored.

Methods: Using freshly isolated skin fibroblasts from RTT patients and healthy subjects, we have demonstrated in RTT cells high levels of H2O2 and HNE protein adducts.

Results: These findings correlated with the constitutive activation of NADPH-oxidase (NOX) and that was prevented by a NOX inhibitor and iron chelator pre-treatment, showing its direct involvement. In addition, also the mitochondria are affected in RTT. In particular, we demonstrated a reduced mitochondrial bioenergetics coupled with an increased mitochondrial oxidant production. The molecular pathways of mitochondrial biogenesis and the fusion/fission dynamics are also impaired as demonstrated by altered levels of mithofusin1-2, Park and OPA-1. Further, we found that the activity of the key cellular defensive enzymes, namely glutathione peroxidase, superoxide dismutase and thioredoxin reductases, were also significantly lower in RTT. Finally, RTT cells shown compromised proteasome activity.

Conclusion: Taken all together, our findings suggest that the systemic redox imbalance in RTT can depend from both the pro-oxidant enzyme activation as well as by the mitochondrial dysfunction and the decreased activity of defensive enzymes. Due to these defective processes and also the proteasome activity impairment, the consequent accumulation of post-translational oxidatively modified proteins can lead to cell damage with systemic implications in RTT.

Music makes a difference

Bergström-Isacsson M.

Swedish Rett Center

Rett Syndrome is one of the few diagnoses where the original medical authority, Andreas Rett (as early as 1966), reported music as a necessity and as a need for this population — but what difference does it make? This presentation will give you some examples for how to use music in an everyday perspective, and how it can be applied more specifically as a therapeutic intervention. Research to date provides support for music preference and the importance of music in communication and learning situations for individuals with Rett Syndrome. This paper will connect the general biological human responses to music to the value of music for persons with Rett syndrome. The presenter will illustrate different occasions where musical interventions are important for the results with the help of video excerpts from clinical work.

Mutation spectrum in the *MECP2* gene in patients with Rett syndrome from Russian Federation.

Beskorovaynaya T.S., Zabnenkova V.V., Polyakov A.V.

Research Centre for Medical Genetics, Moscow

Introduction: Rett syndrome (RTT) is a frequent X-linked dominant neurodevelopmental disorder affecting almost

exclusively girls. It is on the second place among mental retardations in female after Down's syndrome [1]. It is characterized by regression and loss of acquired the self-help skills, speech, social contacts and stereotypical hand movements at the age after 12-18 months of normal development. RTT is usually caused by mutations in the MECP2 gene which is located at the Xq28 locus and consists of 4 exons.

Materials and methods: The 154 non-relative girls from Russian Federation with RTT were investigated. Direct sequencing and quantitative multiplex ligation-dependent probe amplification analysis (MLPA) were used.

Results: Different mutations were found in 44.8% cases (69 patients) that almost twice lower worldwide detection rate that is about 80-85% [2]. Missense/nonsense mutations account was about 70.6%, small deletions/duplications/insertions - 23.5%, splice site mutations- 5.9%. The vast majority of mutations are localized in exon 4 (94.1%), mostly represented by mutations that lead to premature termination of protein (67.7%). Also one mutation was detected in exon 1 (c.48_55dup8) that contains in protein isoform MECP2_e1 expressing abundant in brain. Nine point mutations in our study were the most frequent (60.2%): p.R168X (10.3%), p.R306C (10.3%), p.R270X (8.8%), p.R255X (8.8%), p.R294X (4.4%), p.T158M (4.4%), p.P225R (4.4%), p.P152R (4.4%), c.806delG (4.4%). Furthermore, seven novel mutations were detected: c.537_538insT, c.717delC, c.822_828dupTGTGGTG, c.898_899insT, c.975_976dupGT, c.1189_1190insT, c.1155_1200del46insTCCACCTG.

The quantitative MLPA analysis carried out for the 30 girls without mutations has revealed a heterozygous gross deletion including exon 3 and 4 in one case only (3.3%, that much lower than frequency described in classical RTT (38%) [2]).

Conclusions: Found mutation spectrum corresponds to the world data. We suppose the low detection rate of RTT in Russia is explained by the lack of awareness of physicians about Rett syndrome.

References:

[1] Kim IJ, Kim YJ, Son BH, Nam SO, Kang HC, Kim HD, et al. Diagnostic mutational analysis of MECP2 in Korean patients with Rett syndrome. Exp Mol Med 2006; 38:119–25.

[2] Archer HL, Whatley SD, Evans JC, Ravine D, Huppke P, Kerr A, et al. Gross rearrangements of the MECP2 gene are found in both classical and atypical Rett syndrome patients. J Med Genet 2006;43:451–45

PROCEEDINGS S20

Discovery of new genes in Rett syndrome patients by WES

Vidal Falcó S.[1], Lucariello M.[2], Vidal Ocabo E.[2], Brandi Tarrau N.M.[3], Gerotina Mora E.[1], Jurado P.[2], Esteller Badosa M.[2,4], Pineda Marfa M.[1], Armstrong Morón J.[3]

[1]Fundación Sant Joan de Déu, Barcelona, Spain [2]Programa d'Epigenetica i Biologia del Cancer (PEBC), Institut d'Investigació Biomèdica de Bellvitge (IDIBELL), Barcelona, Spain [3]Servicio de Genética Bioquímica&Rett, Hospital Universitari Sant Joan de Déu. Barcelona, Spain [4]Departament de Ciencies Fisiològiques II, Facultat de Medicina, Universitat de Barcelona, Institució Catalana de Recerca i Estudis Avançats (ICREA), Barcelona, Spain.

Introduction: Rett syndrome (RTT) is a developmental disorder of early onset, genetic basis, dominant inheritance and X-

linked. There are described three genes that cause RTT: MECP2, CDKL5 and FOXG1. However, the etiology of 15% of RTT patients still remains unknown. Thus, the aim of this project is to identify new candidate genes in a cohort of patients with RTT phenotype without genetic diagnosis by Whole Exome Sequencing (WES).

Material and Method: The patient and healthy parents without genetic diagnosis and negative CGHarray Cytoarray Plus (180K) (Agilent Microarrays) were analyzed by WES with TruSeq Sample Preparation Kit (Illumina). The filtering criteria used were: search mutations with 1000g MAF below 0.05 in genes with dominant inheritance, de novo, X-linked, autosomal subject to imprinting and/or with functional impact in the CNS. For the validated mutations in genes related with gabaergic pathways (SLC6A1 and GABBR2), we performed RT-qPCR (TaqMan Gene Expression) and Western Blotting assay of RNA and protein extraction from peripheral blood.

Results: Most of the validated mutations are genes expressed in the central nervous system: ion channels and GABA/glutamate/acetylcholine pathways. The preliminary studies of the SLC6A1 and GABBR2 expression were not conclusive.

Conclusions: We do not only identify 1 gene which causes RTT-like phenotype. Pathway of genes has to be address to understand overlapping phenotype, instead to one disease only. Although blood tissue has convenient extraction, we cannot detect RNA and protein of these genes. Our next studies are performing RT-qPCR and Western Blotting assays with RNA and protein extraction from fibroblasts.

NGS in the diagnosis of Rett syndrome

Akimova I.A.[1,3], Konovalov F.A.[1,2], Kanivets I.V.[1,2], Pyankov D.V.[1,2], Dadali E.L.[1,2,3], Korostelev S.A.[1,2]

[1]*Genomed Ltd., Moscow, Russia* [2]*Research Centre for Medical Genetics, Moscow, Russia* [3] *Pirogov Russian National Research Medical University, Moscow, Russia*

Background: Rett syndrome (RTT, MIM 312750) is a progressive neurodevelopmental disorder and one of the most common causes of mental retardation in females, with an incidence of 1 in 10,000–15,000 [1]. Causes of this syndrome are mutations and deletions in MECP2. Comprehensive genetic screening programs have led to the identification of pathogenic methyl-CpG-binding protein 2 (MECP2) mutations in up to 95% of classical Rett syndrome (RTT) patients [2]. Among other techniques point mutations in this gene can be detected by NGS.

Methods: A group of patients with diagnosis “Rett syndrome” or “Rett-like syndrome” was analyzed by high-resolution chromosome microarray analysis (CMA) and next generation sequencing (NGS). These patients were divided to groups depending on molecular-genetic cause of the disease. Exome sequencing was performed on Illumina NextSeq 500 platform. We use TruSightOne V1,1 for the targeted DNA enrichment. Data processing was carried out using own custom pipeline built on open-source components and public databases. OMIM, UCSC PubMed and others were used as reference databases to determine potential genotype-phenotype correlations.

Results: Among the 23 patients with diagnosis “Rett syndrome” or “Rett-like syndrome” there are 3

with point mutation in *MECP2*, 2 - with point mutation in *CDKL5*, 1 - with point mutation in *CLN6*, 1 – with microdeletion – arr Xq28(153,297,497-153,341,626)x1.

Conclusion: There are many genetic causes of Rett-like phenotype. NGS allows not only detect mutations in MECP2 gene, but also in other genes, associated with similar phenotype.

References:

[1] Amir, R. E., Van den Veyver, I. B., Wan, M., Tran, C. Q., Francke, U., Zoghbi, H. Y. Rett syndrome is caused by mutations in X-linked MECP2, encoding methyl-CpG-binding protein 2. Nature Genet. 23: 185-188.

[2] Hardwick, S. A., Reuter, K., Williamson, S. L., Vasudevan, V., Donald, J., Slater, K., Bennetts, B., Bebbington, A., Leonard, H., Williams, S. R., Smith, R. L., Cloosterman, D., Christodoulou, J. Delineation of large deletions of the MECP2 gene in Rett syndrome patients, including a familial case with a male proband. Europ. J. Med. Genet. 15: 1218-1229

PROCEEDINGS S22

Partnerships in care for Rett Syndrome

Budden S.

Providence St Vincent's Hospital Portland Oregon USA

Rett syndrome (RTT) is a well-recognized neuro-developmental disorder predominantly diagnosed in females and resulting from MECP-2 mutations although males have been reported. It is unique in that after a period of early developmental regression there is stabilization with some improvement of function. Immaturity of the brain is supported by neuro-pathological studies. Cortical dysfunction effects cognition, communication and motor function. Pervasive effects on autonomic nervous system are evident in respiratory dysfunction

gastro-intestinal system, sleep and vasomotor effects. Behaviour disturbances and sudden unexplained death syndrome may occur as a result of brain stem serotonergic deficiency. Bone growth is affected and muscle development is poor. RTT is not a degenerative condition and potential for functional development is difficult to measure since the expression of MECP2 mutations on neurobiological mechanisms change with age. Brain immaturity provides opportunities to encourage development of new skills and maintain current function. There is compelling evidence showing that intervention in enriched environments maximizes abilities and facilitate emerging skills. Caring for individuals with complex multiple medical conditions physicians are challenged in providing continuity of coordinated care. Creating partnerships provides opportunities to share current information on the biological mechanisms impacting individuals with RTT with primary physicians, subspecialists, therapists, social services, educational systems and parents or care givers as important members of a team. Partnerships should be child and family-centred, community based, comprehensive, continuous, collaborative, compassionate, communicative and culturally competent. The overarching principle in rehabilitation is maintaining medical stability and providing continuity of care. Assessments include identification for seizures, sleep disorders, respiratory problems, dysphagia, reflux, constipation, scoliosis, osteoporosis and causes of agitation and behavioural dysfunctions. Careful documentation of growth, dental

status, vision and hearing, EKGs for long QTc and peripheral autonomic disturbances is essential An integrated team approach focusing on assessment and management of maintaining motor function and preventing deformities of hips, spine and feet and identifying fractures is crucial in maintaining mobility. Activities of daily living (ADL) include feeding and augmentative communication. Educational curriculum includes motor therapies, Augmentative Communication, Music Therapy and Psychosocial support. Caring for adults with RTT is a challenge since therapy interventions and monitoring become infrequent as the women age and should be addressed. Enriched environments and ability to access activity centres provide opportunities to become assimilated within society. Continuity of good medical psychosocial support and therapeutic involvement remains a major challenge for all.

References:

[1] Budden SS, Gunness ME. (2003) Possible mechanisms of osteopenia in Rett syndrome. Bone histomorphometric studies .J Child Neurol 18(10): 698-702.

[2] Motil KJ, Shultz RJ, Glaze DG, Armstrong D. (2001) Oroparyngeal dysfunction and Upper gastrointestinal dysmotility, a reflection of disturbances in the autonomic- nervous system in Rett syndrome. In Rett disorder and the Developing Brain, Kerr A and Witt-Engerstrom I (Eds) Oxford University Press, Oxford p 259.

[3] Kondo M, Gray LJ, Pelka GJ, Christodoulou J, Tam PPL, Hannan AJ. (2005) Environmental enrichment ameliorates a motor coordination deficit in mouse model of Rett syndrome –Mecp2 gene dose effects and BDNF exposure, European J of Neuroscience 27: 3342-3350

[4] Lotan M, Hanks SB,(2006) Physical therapy interventions for individuals with Rett Syndrome. The Scientific World Journal 6:1314-1338

Rett syndrome phenotype is modulated by an X-linked parent-of-origin-like effect: evidences from molecular cytogenetic analysis and data on *MECP2* mutations and X chromosome inactivation patterns

Iourov I.Y.[1,2,3], Vorsanova S.G.[1,2,4], Voinova V.Y.[1,2,4], Demidova I.A.[1,2,4], Kolotii A.D.[1,2], Kravets V.S.[1,2,4], Kurinnaia O.S.[1,2,4], Zelenova M.A.[1,2,4], Soloviev I.V.[1], Yurov Y.B.[1,2,4]

[1]Mental Health Research Center, Moscow, Russian Federation [2]Research and Clinical Institute for Pediatrics named after Y.E. Veltishev at the Pirogov Russian National Research Medical University, Ministry of Health, Moscow, Russian Federation [3]Department of Medical Genetics, Russian Medical Academy of Postgraduate Education, Ministry of Health, Moscow, Russian Federation. [4]Moscow State University of Psychology and Education, Moscow, Russian Federation

Regardless of significant efforts made throughout the last two decades, the pathway from molecular pathology to phenotype (i.e. genotype/phenotype correlations) in Rett syndrome (RTT) has not been presented [1, 2]. In addition to commonly considered phenotype modulators in RTT (*MECP2* mutation position/type and X chromosome inactivation patterns), it has been previously suggested that parental origin of predominantly inactivated chromosome X would contribute to phenotypic variability in RTT [3]. Lately, the suggestion was partially confirmed [4]. Here, a detailed description of the study is given.

Studying the RTT cohort [4, 5], we have detected *MECP2* mutations in 84.6% of girls. It is noteworthy that 5 mutations were unique to this cohort. Analyzing X chromosome inactivation patterns [3, 4], it was shown that skewed X-inactivation is observed in 37% of

RTT girls (significantly higher than in controls; 6.5%). DNA replication patterns revealed by cytogenetic analysis of the type C replication and fluorescence *in situ* hybridization evaluation of chromosomal DNA replication at Xq28 correlated with X chromosome inactivation patterns showed the epigenetic abnormalities to result from *MECP2* mutations. Addressing X chromosome inactivation in RTT girls and their mothers, we found that a kind of parent-of-origin effect does affect the phenotype in this disease. Firstly, the direction of X-inactivation skewing (i.e. inactivation of either paternal or maternal chromosome X) modulates the phenotypic outcome of a *MECP2* mutation leading to milder forms because of paternal X chromosome skewing and to more severe forms because of maternal X chromosome skewing. Furthermore, since *MECP2* mutations have been shown likely to result in epigenetic abnormalities affecting the X chromosome behavior, an indirect mechanism for the phenotypic outcome may be proposed. More precisely, the most probable one is an X-linked parent-of-origin-like effect.

To test whether an X-linked parent-of-origin-like effect is able to modulate RTT phenotype, we have addressed cases of Xq28 deletions causing RTT in presumably *MECP2*-mutation-negative cases described previously [5-7]. It has been observed that a deletion does not result in X chromosome inactivation skewing. Although alterations to chromosome X causing the so-called X-linked dominant conditions are considered to be likely cause for X chromosome inactivation skewing [1-4], this is not a case for Xq28 (*MECP2*) deletions. Thus, in

contrast to canonical mechanisms for X chromosome inactivation skewing, RTT exhibits a previously unknown X-linked parent-of-origin-like effect originating from *MECP2* mutations or copy number variations modulating as milder as more severe RTT cases. Therefore, to correlate RTT phenotype and genotype, one has to consider the direction of X-inactivation skewing, indirect action of *MECP2* mutations or copy number variations on X chromosome inactivation and related epigenetic phenomena (i.e. DNA replication), and cumulative X-linked parent-of-origin-like effect. This study was supported by the Russian Science Foundation (project #14-15-00411).

References:

[1] Vorsanova SG, Iourov IY, Yurov YB. Neurological, genetic and epigenetic features of Rett syndrome. J Pediatr Neurol. 2004; 2(4):179-190.

[2] Halbach NS, Smeets EE, van den Braak N, van Roozendaal KE, Blok RM, Schrander-Stumpel CT, Frijns JP, Maaskant MA, Curfs LM. Genotype-phenotype relationships as prognosticators in Rett syndrome should be handled with care in clinical practice. Am J Med Genet A. 2012; 158A(2):340-50.

[3] Yurov YB, Vorsanova SG, Kolotii AD, Iourov IY. Molecular-cytogenetic investigation of skewed chromosome X inactivation in Rett syndrome. Brain Dev.2001;23 Suppl1:S214-7.

[4] Iurov IIu, Vorsanova SG, Voinova-Ulas VIu, Villard L, Demidova IA, Giunti L, Guivabyccu-Uzielli ML, Budilov AV, Beresheva AK, Novikov PV, Iurov IuV. [Epigenetic study of Rett's syndrome as an adequate model for autistic disorders]. Zh Nevrol Psikhiatr Im S S Korsakova. 2005; 105(7):4-11.

[5] Iourov IY, Vorsanova SG, Voinova VY, Kurinnaia OS, Zelenova MA, Demidova IA, Yurov YB. Xq28 (*MECP2*) microdeletions are common in mutation-negative females with Rett syndrome and cause mild subtypes of the disease. Mol Cytogenet. 2013; 6(1):53.

[6] Iourov IY, Vorsanova SG, Kurinnaia OS, Zelenova MA, Silvanovich AP, Yurov YB. Molecular karyotyping by array CGH in a Russian cohort of children with intellectual disability, autism, epilepsy and congenital anomalies. Mol Cytogenet. 2012; 5(1):46.

[7] Vorsanova SG, Iurov IIu, Voinova VIu, Kurinnaia OS, Zelenova MA, Demidova IA, Ulas EV, Iurov IuB. [Subchromosomal microdeletion identified by molecular karyotyping using DNA microarrays (array CGH) in Rett syndrome girls negative for *MECP2* gene mutations]. Zh Nevrol Psikhiatr Im S S Korsakova. 2013; 113(10):63-8.

Phosphorylation of huntingtin at Ser421 modulates the phenotype of the Mecp2 deficient mice.

Ehinger Y., Saidi L., Matagne V., Bruyère J., Saudou F., Villard L., Roux J.C.

UMR_S910, Medical Genetics and Functional Genomics, Marseille.

Rett syndrome (RTT) is a neurological disorder caused by the X-linked MECP2 gene mutation. Previously, we demonstrated that the Huntingtin-dependant (Htt) axonal transport is altered when Mecp2 is lacking, partly due to a deficit of the molecular motor contents (Roux et al., 2012). However, the neuronal trafficking is also strongly dependent on the phosphorylation level of Htt at serine 421 (S421). Therefore, we developed several tools in order to stimulate pharmacologically Htt phosphorylation at S421 in vivo and in vitro using: 1) a direct activation of the Akt pathway, through the stimulation of the insulin/IGF1 receptors or, 2) the indirect blocking of the Htt dephosphorylation using Fk506. Thereafter, we used a genetic approach by crossing Mecp2 deficient mice with knock in mice expressing either an aspartic acid or alanine at position 421 to mimic tonic phosphorylation (S421D) or to prevent phosphorylation (S421A), respectively. For both pharmacology and genetic crossing we used a battery of behavioral tests in order to evaluate the in vivo consequence: grip strength, rotarod, open field and the respiratory profile during postnatal development are performed. Altogether our results

indicate that modulation of Htt at serine 421 is a promising way to improve the neuronal trafficking in RTT and a possible target to develop treatments.

Rett Syndrome as a result of aberrant DNA methylation: an epigenetic transgenerational inheritance of epimutation and its expression

Klymnyuk S.I.[1], Kovanova E.N.[2], Tvorko M.S.[3], Pokryshko E.V.[4], Bihunyak T.V.[5]

[1]Head of the Microbiology, Virology and Immunology Department SHEI "Ternopil State Medical University by I.Ya. Horbachevsky", Ukraine [2,3,4]Associates Professor of the Microbiology, Virology and Immunology Department SHEI "Ternopil State Medical University by I.Ya. Horbachevsky", Ukraine 5Associate Professor of the Medical Biology Department SHEI "Ternopil State Medical University by I.Ya. Horbachevsky", Ukraine

Rett syndrome (RS) is an X-linked inherited disease that is more common in girls and is caused by mutations *de novo*. The main reason of the RS is considered to be the mutations of gene *MECP2* (*methyl CpG binding protein 2*) [1]. Phenotype of RS without changing the genotype is observed in girls, as well as rare cases of disease in males can be explained by epigenetic changes of *MECP2* gene associated with aberrant DNA methylation at *CpG* islands – epimutations *C→mC*. In girls, this epimutation expresses in heterozygous alleles position of X chromosome of paternal (*pat*) and maternal (*mat*) origin. Boys' expression of epimutations *C→mC* is due to the presence of defects in monogenic recessive homologous

portions *q28* X and Y chromosomes. Boys can express aberrant methylation of *pat* and *mat* alleles in the F2 generation.

If methyl-cytosine (*mC*) is deaminated spontaneously without enzyme and converted into thymine pair *C-G* in DNA replication is replaced by *A-T* pair [2], thereby epimutation is converted to a mutation, resulting in impaired replication transcription and translation of DNA, modified amino acid codons which leads to deregulation of the gene work. Stop codons *Ochre*, *Amber* and *Opal* can lose their termination function and can encode amino acids, which leads to the disruption of protein synthesis *MeCP2*. Replacement of *C-G* to *A-T* cytosine amino acid codons may lead to the acquisition of the properties in the initiation of DNA transcription (instead of methionine).

Thus, epimutations and substitution mutations in RS, which are induced by environmental factors, in girls are associated with X chromosomes, and in the boys – with X and Y chromosomes.

References:

[1] Christodoulou J., Ho G. MECP2-Related Disorders // Gene Reviews [Internet]. Seattle (WA): University of Washington, Seattle; 1993-2015. 2001 [updated 2012]. PMID: 20301670.

[2] Gvozdev V.A. Reguljacija aktivnosti genov, obuslovlennaja himicheskoj modifikaciej (metilirovaniem) DNK / V.A. Gvozdev // Sorosovskij obrazovatel'nyj zhurnal. 1999. – № 10. – S. 11-17.

Rett syndrome journeys: What Russia and the world can learn from the experience of a population-based and an international registry of Rett syndrome.

Leonard H.

Telethon Kids Institute in Western Australia

In 1993 there was minimal published information in Australia or elsewhere about the epidemiology of the rare neurodevelopmental disorder Rett syndrome or about many rare disorders. The cause of Rett syndrome was also unknown. In response to this need, I established and have successfully maintained the first population-based database of this disorder. These data have been used to inform the understanding of Rett syndrome including providing best estimates of life expectancy based on 20 years of longitudinal data.

After the genetic cause of Rett syndrome was identified in 1999, it also became clear that even larger data samples would be needed to study the effects of the genetic variability in this disorder. This was the impetus for the establishment of an international Rett syndrome database in 2002.

Using both database structures we have been able to demonstrate clear genotypic differences in the presentation of various comorbidities, which have important downstream implications for clinical management. Furthermore, using available literature and input from international expert panels, we have now developed guidelines for the management of several comorbidities in Rett syndrome.

This presentation will include information about: diagnosis; the

range of phenotypes and how they are influenced by genotype; several of the major comorbidities that affect girls and women with Rett syndrome and the guidelines we have developed to manage these comorbidities.

Rett syndrome molecular defects are associated with specific epigenetic alterations at chromosomal level

Vorsanova S.G.[1,2,3], Yurov Y.B.[1,2,3], Demidova I.A.[1,2,3], Kolotii A.D.[1,2], Kravets V.S.[1,2,3], Soloviev I.V.[2], Iourov I.Y.[1,2,4]

[1]Research and Clinical Institute for Pediatrics named after Y.E. Veltishev at the Pirogov Russian National Research Medical University, Ministry of Health, Moscow, Russian Federation [2]Mental Health Research Center, Moscow, Russian Federation [3]Moscow State University of Psychology and Education, Moscow, Russian Federation [4]Department of Medical Genetics, Russian Medical Academy of Postgraduate Education, Ministry of Health, Moscow, Russian Federation.

Rett syndrome (RTT) has been repeatedly shown to demonstrate reproducible pattern of epigenetic alterations [1]. However, these alterations have been rarely studied at the supramolecular or chromosomal level. Nonetheless, late-replicating chromosomes X featured by a specific banding pattern (type C) with unusual staining of chromatin at the X chromosome long arm are observed almost exclusively in RTT females suggesting that there are epigenetic alterations (i.e. altered chromatin conformation in inactive chromosome X) specific for RTT [2, 3]. Furthermore, similar types of epigenetic alterations can segregate with a phenotype [4, 5]. Here, we have attempted to determine whether

epigenetic alterations observed at chromosomal level are associated with molecular pathology and epigenetic changes (X chromosome inactivation addressed by molecular genetic techniques) in RTT.

Correlating data on *MECP2* mutations and X chromosome inactivation patterns [6] with epigenetic alterations detectable at chromosomal level has shown the latter to result from molecular RTT pathology. *MECP2* mutations typical for RTT (recurrent nonsense and T158M) were associated with epigenetic alterations observed at chromosomal level. The amount of cells (metaphase plates) featured by these alterations positively correlated with X chromosome inactivation skewing. More precisely, the higher were degrees of skewing (extreme skewing of X chromosome inactivation) the commoner was the occurrence of cells with abnormal replication and chromatin conformation of inactivated chromosome X. Furthermore, rare cases of RTT caused by Xq28 deletions have exhibited these epigenetic alterations. Consequently, phenotypic outcome is likely to be a result of epigenetic disturbances in X chromosome behavior and organization. Thus, RTT epigenetic alterations at chromosomal level are an important element of RTT pathogenic pathway with a relevance to the phenotype.

To explain the RTT epigenetic pathology, we have proposed a hypothesis linking *MECP2*, X-inactivation and altered replication/chromatin conformation. The hypothesis is based on observations depicting *MECP2* involvement in LINE-1 retrotransposition [7]. Accordingly, mutations may lead to instability of transcriptional

repression of LINE-1 retroelements, which can be a cause of an incomplete transcriptional X chromosome inactivation and replication due to expression of genes which should have been inactive. LINE-1 are conservative elements of the genome presented as dispersed repeats which should normally be inactivated. Activation and expression of these repeats may lead to an aberrant DNA methylation causing skewed X chromosome inactivation, and abnormal replication and chromatin conformation of inactivated chromosome X. Certainly, additional studies are required to test the hypothesis. Nevertheless, this study provides evidences that RTT molecular defects are associated with specific epigenetic alterations at chromosomal level. The study was supported by the Russian Science Foundation (project #14-15-00411).

References:

[1] Pohodich AE, Zoghbi HY. Rett syndrome: disruption of epigenetic control of postnatal neurological functions. Hum Mol Genet. 2015; 24(R1):R10-6.

[2] Vorsanova SG, Demidova IA, Ulas VYu, Soloviev IV, Kazantzeva LZ, Yurov YuB. Cytogenetic and molecular-cytogenetic investigation of Rett syndrome: analysis of 31 cases. Neuroreport. 1996; 8(1):187-9.

[3] Vorsanova SG, Yurov YB, Ulas VY, Demidova IA, Sharonin VO, Kolotii AD, Gorbatchevskaia NL, Beresheva AK, Soloviev IV. Cytogenetic and molecular-cytogenetic studies of Rett syndrome (RTT): a retrospective analysis of a Russian cohort of RTT patients (the investigation of 57 girls and three boys). Brain Dev. 2001; 23 Suppl 1:S196-201.

[4] Vorsanova SG, Voinova VIu, Iurov IIu, Kurinnaia OS, Demidova IA, Iurov IuB. [Cytogenetic, molecular cytogenetic, clinical and genealogical study of mothers of children with autism: a search for family genetic markers of autistic disorders]. Zh Nevrol Psikhiatr Im S S Korsakova. 2009; 109(6):54-64.

[5] Vorsanova SG, Voinova VY, Yurov IY, Kurinnaya OS, Demidova IA, Yurov YB. Cytogenetic, molecular-cytogenetic, and clinical-genealogical studies of the mothers of children with autism: a search for familial genetic markers for autistic

disorders. Neurosci Behav Physiol. 2010; 40(7):745-56.
[6] Iurov IIu, Vorsanova SG, Voinova-Ulas VIu, Villard L, Demidova IA, Giunti L, Guivabyccu-Uzielli ML, Budilov AV, Beresheva AK, Novikov PV, Iurov IuV. [Epigenetic study of Rett's syndrome as an adequate model for autistic disorders]. Zh Nevrol Psikhiatr Im S S Korsakova. 2005; 105(7):4-11.
[7] Muotri AR, Marchetto MC, Coufal NG, Oefner R, Yeo G, Nakashima K, Gage FH. L1 retrotransposition in neurons is modulated by MeCP2. Nature. 2010; 468(7322):443-6.

Russian Rett syndrome cohort: clinical and molecular characterization

Voinova V.Y.[1,2,3], Vorsanova S.G.[1,2,3], Yurov Y.B.[1,2,3], Demidova I.A.[1,2,3], Iourov I.Y.[1,2,4]

[1]Research and Clinical Institute for Pediatrics named after Y.E. Veltishev at the Pirogov Russian National Research Medical University, Ministry of Health, Moscow, Russian Federation [2]Mental Health Research Center, Moscow, Russian Federation [3]Moscow State University of Psychology and Education, Moscow, Russian Federation [4]Department of Medical Genetics, Russian Medical Academy of Postgraduate Education, Ministry of Health, Moscow, Russian Federation.

During the last 25 years, 16.292 girls with intellectual disability and congenital malformations were observed in the Department of Clinical Genetics. Among them, 391 individuals had Rett syndrome (RTT). Consequently, RTT frequency has been estimated as 2.4% of girls with intellectual disability. For more details, please refer to our previous cytogenetic, molecular and clinical descriptions of Russian RTT cohort [1-7].

Currently, Russian RTT cohort includes 394 patients: 3 males and 391 females (age: from 22 months to 32 years). Genealogically, the majority of cases were shown to be sporadic. There is a monozygotic twin pair and a familial case (maternal half-/.lm).

The total sibling number is 122 (54 brothers and 68 sisters). There are siblings with mild developmental delay (4 brothers and 2 sisters), speech disorder (2 brothers), hyperactivity (1 sister) and autism with epilepsy (1 case of paternal half-sister); 90 out of 122 siblings (74%) were born before RTT child and 32 (26%) — after RTT child indicating frequent parental refusal to have additional children. There are 301 classic and 93 atypical RTT patients (39 children – 'forme fruste', 17 – early-onset seizures, 16 - congenital, 12 - preserved speech, 12 - late regression variant). Three boys had classic RTT. *MECP2* gene mutations were studied in 354 individuals. Most of classic (249/ 262; 95%) and 65 of 92 of atypical (70,7%) cases have been attributed to *MECP2* mutations. Recurrent point mutations were identified in 201 (64%) cases. The most common was R255X (50 cases; 16%), including twins pair. The occurrence of other recurrent mutations was 3% (R106W), 7% (R133C), 14% (T158M), 11% (R168X), 9% (R270X), 3% (R294X), and 8% (R306C). One boy had somatic mosaicism for R270X mutation, whereas the other had the R168X mutation and tissue-specific chromosomal mosaicism 46,XY/47,XXY [3]. *CDKL5* gene mutations were found in four girls with early-onset seizure variant. In *CDKL5* and *MECP2*-mutation-negative patients, 6 classic and 5 atypical RTT cases showed Xq28 (*MECP2)* deletions and 2 cases exhibited *CDKL5* deletions [6, 7]. Genotype/phenotype correlations confirmed that RTT clinical heterogeneity likely involves a complex interplay between numerous factors, including mutation type/position and X-chromosome inactivation

patterns. However, it is not always possible to link individual phenotypical variability with molecular data, mainly due to difficulties of assessing intertissular differences of X-chromosome inactivation patterns. Nevertheless, molecular data allow prognosis in significant number of cases. The study was supported by the Russian Science Foundation (project #14-15-00411).

References:

[1] Vorsanova SG, Demidova IA, Ulas VYu, Soloviev IV, Kazantzeva LZ, Yurov YuB. Cytogenetic and molecular-cytogenetic investigation of Rett syndrome: analysis of 31 cases. Neuroreport. 1996; 8(1):187-9.

[2] Vorsanova SG, Ulas VIu, Demidova IA, Kravets VS, Iurov IuB. [Contemporary views on Rett's syndrome: clinical, cytogenetic and molecular studies]. Zh Nevrol Psikhiatr Im S S Korsakova. 1999; 99(3):61-9.

[3] Vorsanova SG, Yurov YB, Ulas VY, Demidova IA, Sharonin VO, Kolotii AD, Gorbatchevskaia NL, Beresheva AK, Soloviev IV. Cytogenetic and molecular-cytogenetic studies of Rett syndrome (RTT): a retrospective analysis of a Russian cohort of RTT patients (the investigation of 57 girls and three boys). Brain Dev. 2001; 23 Suppl 1:S196-201.

[4] Vorsanova SG, Ulas VIu, Iurov IuB, Giovanucci-Uzielli ML, Demidova IA, Gianti L, Villard L, Iurov IIu, Beresheva AK, Novikov PV. [Genotype-phenotype correlations in Rett syndrome: the study of Russian cohort of patients]. Zh Nevrol Psikhiatr Im S S Korsakova. 2002; 102(10):23-9.

[5] Iurov IIu, Vorsanova SG, Voinova-Ulas VIu, Villard L, Demidova IA, Giunti L, Guivabyccu-Uzielli ML, Budilov AV, Beresheva AK, Novikov PV, Iurov IuV. [Epigenetic study of Rett's syndrome as an adequate model for autistic disorders]. Zh Nevrol Psikhiatr Im S S Korsakova. 2005; 105(7):4-11.

[6] Iourov IY, Vorsanova SG, Voinova VY, Kurinnaia OS, Zelenova MA, Demidova IA, Yurov YB. Xq28 (*MECP2*) microdeletions are common in mutation-negative females with Rett syndrome and cause mild subtypes of the disease. Mol Cytogenet. 2013; 6(1):53.

[7] Vorsanova SG, Iurov IIu, Voinova VIu, Kurinnaia OS, Zelenova MA, Demidova IA, Ulas EV, Iurov IuB. [Subchromosomal microdeletion identified by molecular karyotyping using DNA microarrays (array CGH) in Rett syndrome girls negative for *MECP2* gene mutations]. Zh Nevrol Psikhiatr Im S S Korsakova. 2013; 113(10):63-8.

The electroencephalographic (EEG) features in children with Rett syndrome

Kvaskova N.E., Abramov M.O., Ivanova I.V., Mukhin K.Yu.

Svt. Luka's Institute of Child Neurology and Epilepsy, Moscow

Rett syndrome, is a progressive usually sporadic neurogenetic disorder with onset in early childhood predominantly affecting females, characterized by psychomotor regression, autistic behavior, loss of purposeful hand skills, specific hand stereotypies and often a seizure disorder (Mukhin K.Yu. et al, 2014).

Purpose: to study the electroencephalographic (EEG) characteristics of patients with Rett syndrome.

Materials and Methods: Thirteen female patients with genetically proven Rett syndrome (MECP2 mutation) and epilepsy aged from 1 year 9 months to 11 years (mean age 6,5±3,7 years) were included in this study. All patients were performed prolonged video-EEG monitoring during both wakefulness and sleep, International 10-20 system.

Results: video-EEG monitoring revealed: slowing of the background activity in 10 patients (77%); normal background with sustained physiological sleep and wake patterns in 3 girls (23%) less than or equal to 2 years of age. In 31% of cases sleep and wake state were poorly differentiated with fragmented physiological sleep patterns. Twelve patients (92%) had periodic regional slowing most often localized in central-temporal (38%), fronto-central (23%), occipital (23%) and temporal (15%) regions. In addition to

regional changes EEG revealed bilateral slowing in fronto-central (54%), frontal (31%), occipital (23%) and centro-parieto-temporal (8%) areas. In 8 patients (61%) the rhythmical theta activity (4-5/sec or 5-6/sec) over the fronto-central region was present in waking state. Epileptiform abnormalities (EA), including spikes, sharp waves, spike-and-wave, sharp and slow-wave complexes, were present in all patients. In 2 patients (15%) under 3 years of age epileptiform activity were not present during waking state. EA included: regional discharges in 3 patients (23%); multiregional associated with diffuse discharges in 4 cases (31%); in the remaining patients (46%) regional or multiregional EA were seen in different associations with bisynchronous or lateralized discharges of spike-wave, sharp and slow-wave complexes.

In most cases (69%) epileptiform abnormalities were localized in the central regions, appeared in groups and had morphology similar to Benign Focal Epileptiform Discharges in Childhood (BFEDC) - "Rolandic spikes". These patients had aggravation of EA during sleep.

Conclusions: EEG in the patients with Rett syndrome is characterized by a progressive deterioration with slowing of background activity, loss of normal wake and sleep characteristics, and the appearance of regional, then multiregional, and diffuse epileptiform abnormalities. In some patients with Rett syndrome the peculiar EEG pattern of rhythmical theta activity (4-5/sec or 5-6/sec) over the central regions was present. The similar pattern was mentioned by H. Doose (2003) in epilepsies with "genetic predominance". In the

patients with Rett syndrome epileptiform abnormalities were often localized in central regions with morphology similar to BFEDC, and evolution of these EEG characteristics resemble electrographic features of idiopathic focal epilepsies.

The method of resonant co-creation (MRC: Resonante Cokreation, Sibgatullina I. / Gruessl S., 2002) in the complex rehabilitative care for children with Rett syndrome

Sibgatullina I.F.

RBS- Privatinstitut zur Foerderung der intelletuellen Entwicklung RBS-IFIE

The method of resonant co-creation (MRC: Resonante Cokreation, Sibgatullina I. / Gruessl S., 2002) was created, and is continuing to develop, in Russia (Kazan) and Austria (Vienna). The method of resonant co-creation (MRC: Resonante Cokreation, Sibgatullina I. / Gruessl S., 2002) can be applied in the complex rehabilitative care for children with Rett syndrome to establish emotional contact and to stabilize decrease/deceleration rate of mental development regression. The method is based on the natural sciences and psychobiological understanding of resonance (French: résonance, Latin: resono – to resonate) as a phenomenon of a sharp increase in the amplitude of the forced oscillations, which occurs when frequency of external influence approaches to certain values (resonant frequencies) determined by attributes of the system. With the resonance it is possible to strengthen even very weak vibrations and to distinguish

the subject of conditional creative activity of children with Rett syndrome. Possible detected stable values of creativity are minimal, but they have quite a positive effect on the overall development of social and mental skills. Artistic image has a special mission in the method of resonant co-creation (MRC: Resonante Cokreation, Sibgatullina I. / Gruessl S., 2002). The method is based on the phenomenon of selective perception of images of children with Rett syndrome. Artistic image acts as a stimulus/driving force and the perception acts as, in fact, the oscillating system. In work with children with Rett syndrome, a resonance phenomenon is that at a certain frequency of the driving force of considering bright artistic image oscillating system of the brain activity is particularly responsive to the action of this force. The method of resonant co-creation (MRC: Resonante Cokreation, Sibgatullina I. / Gruessl S., 2002) considers relevant to Rett syndrome degree of formed/unformed prefrontal cortex with the advantages of hemispheric activation as the main preconditions of perception of art image/art. The comprehensive assistance to children involves neurologist, a physician-rehabilitator, psychologist and artist and/or art-therapist. Experts join their forces and form a mental growth of children with this rare disease together. Work takes place individually and in short sessions. The linguistic element of the method is minimal, but it varies in consideration of each individual case in practice. Rehabilitation practice of the method (MRC: Resonante Cokreation, Sibgatullina I. / Gruessl S., 2002) takes place systematically and always aims to work with the

artistic image of different content, different forms of expression, and color of the artistic image. For a systematic use of the method, the experts need education and training in the field of comprehensive care for children with rare diseases. Educational program includes theoretical module of studying the method of resonant co-creation (MRC: Resonante Cokreation, Sibgatullina I. / Gruessl S., 2002), module of practical skills and the organization of supervision. Training is delivered by Russian and Australian experts and authors of the method. Methodical art resources have been developing in Austria since 2002.

1q21.1q21.2 duplications can cause in Rett-like phenotype

Yurov Y.B.[1,2,3], Vorsanova S.G.[1,2,3], Zelenova M.A.[1,2,3], Kurinnaia O.S.[1,2,3], Voinova V.Y. [1,2,3], Iourov I.Y.[1,2,4]

[1]Mental Health Research Center, Moscow, Russian Federation [2]Research and Clinical Institute for Pediatrics named after Y.E. Veltishev at the Pirogov Russian National Research Medical University, Ministry of Health, Moscow, Russian Federation [3]Moscow State University of Psychology and Education, Moscow, Russian Federation [4]Department of Medical Genetics, Russian Medical Academy of Postgraduate Education, Ministry of Health, Moscow, Russian Federation.

Genomic rearrangements within 1q21.1 chromosomal region have been associated with various abnormalities such as developmental delay, growth retardation, seizures, heart defects, schizophrenia, abnormal head size (macrocephaly) and facial dysmorphisms [1, 2]. Here, we have studied the cohort of children with intellectual disability, congenital malformations, epilepsy and autism (including 24 individuals with Rett-like phenotype) by molecular karyotyping using Affymetrix Cytoscan HD (2,7M probes) and Nimblegene (180K probes) microarray platforms together with original bioinformatic technology developed for addressing cases of chromosomal or genomic imbalances [3,4]. We have revealed duplications at 1q21.1q21.2 in 5 patients, three of which had a Rett-like phenotype (unnecessary movements, weak reflexes but no “classic” behaviors). The duplications affected three girls and two boys. Atrial fibrillation, atrial standstill and cataract are among the disorders associated with some

genes in this region. Our patients presented with intellectual disability, autistic behavior (4 cases), foot abnormalities (2 cases), epilepsy (1 case), and microcephaly (3 cases). Additionally, partial optical nerve atrophy and ICP were noted in two different patients.

The genomic region shared in these duplication cases was mapped within chromosome 1q21.1q21.2 (146,111,761-148,043,201). The region spans 13 genes indexed in OMIM (online Mendelian inheritance in man): *NBPF12, PRKAB2, FMO5, CHD1L, BCL9, ACP6, GJA5, GJA8, GPR89B, NBPF20, NBPF10, NBPF11, NBPF8*. We have identified 2 candidate genes for Rett-like phenotype and shared phenotypic features in these duplications (i.e. congenital heart anomalies and eye abnormalities): *GJA5* and *GJA8*. According to the literature, 1q21 deletions and duplications have mostly been described as having strabismus and cataract. In our cohort, two patients presented with partial optic nerve atrophy [1, 2]. There were no patients with reported cardiac problems. Interestingly, interactome analysis showed pathways that are likely to be involved in the phenotypic outcome. *CHD1L* encodes a DNA helicase protein involved in DNA repair and *BCL9* is associated with a conferring risk of schizophrenia. It is interesting to note that *BCL9* is implicated in Wnt signaling pathways which are important for axon development and guidance along with synapse formation and maintenance. Interactomic analyses also showed that duplicated genes interact with essential Rett syndrome genes (i.e. *MECP2* and *CDKL5*). Interestingly, both duplications revealed in males were mosaic suggesting some parallels with classical Rett syndrome, which can manifest in

males in cases of somatic mosaicism [5].

In conclusion, this is the first report showing 1q21.1q21.2 duplications to result in Rett-like phenotype. Additional studies are certainly required to identify molecular mechanisms of Rett-like phenotype. This study was supported by the Russian Science Foundation (project #14-35-00060).

References:

[1] Mefford HC, Sharp AJ, Baker C, Itsara A, Jiang Z, Buysse K et al. Recurrent Rearrangements of chromosome 1q21.1 and variable pediatric phenotypes. N Engl J Med. 2008; 359(16):1685-99.

[2] Brunetti-Pierri N, Berg JS, Scaglia F, Belmont J, Bacino CA, Sahoo T et al. Recurrent reciprocal 1q21.1 deletions and duplications associated with microcephaly or macrocephaly and developmental and behavioral abnormalities. Nat Genet. 2008; 40(12):1466-71.

[3] Iourov IY, Vorsanova SG, Kurinnaia OS, Yurov YB. An interstitial deletion at 10q26.2q26.3. Case Rep Genet. 2014; 2014:505832.

[4] Iourov IY, Vorsanova SG, Demidova IA, Aliamovskaia GA, Keshishian ES, Yurov YB. 5p13.3p13.2 duplication associated with developmental delay, congenital malformations and chromosome instability manifested as low-level aneuploidy. Springerplus. 2015; 4:616.

[5] Iourov IY, Vorsanova SG, Yurov YB. Somatic genome variations in health and disease. Curr Genomics. 2010; 11(6):387-96.

PROCEEDINGS S32

3q27.1 microdeletion and Rett-like phenotype

Vasin K.S.[1,2,3], Vorsanova S.G.[1,2,3], Yurov Y.B.[1,2,3], Kurinnaia O.S.[1,2,3], Voinova V.Y.[1,2,3], Iourov I.Y.[1,2,4]

[1]Mental Health Research Center, Moscow, Russian Federation [2]Research and Clinical Institute for Pediatrics named after Y.E. Veltishev at the Pirogov Russian National Research Medical University, Ministry of Health, Moscow, Russian Federation [3]Moscow State University of Psychology and Education, Moscow, Russian Federation [4]Department of Medical Genetics, Russian Medical Academy of Postgraduate Education, Ministry of Health, Moscow, Russian Federation.

Rett Syndrome (RTT) is a neurodevelopmental disorder affecting almost exclusively females (incidence: ~1:15000). It is characterized by an apparently normal early development, followed by developmental stagnation and regression, leading to loss of purposeful hand movements, reduced head and brain growth, physical disabilities, seizures, and intellectual disability. Usually, patients show first symptoms between 6-18 months of age and display cognitive function progressive loss as well as loss of fine and gross motor skills with abnormal social-cognitive development. Apart from *MECP2* mutations, atypical RTT/RTT-like phenotypes can be caused by other single gene mutations, genome unbalances or copy number variations [1, 2]. Array CGH (NimblGen 135K platform) with a bioinformatics algorithm were performed as described previously [2, 3]. In an 8-year-aged girl with RTT-like phenotype without detectable *MECP2* mutation, 249 kb microdeletion at 3q27.1 was revealed. The girl demonstrated arrest in development at 8 months, the second regression of acquired skills at 12-18 months. At the age of 8, she presented with severe intellectual disability, scoliosis, constant stereotypic hand movements, tremor, regular seizures, lost the ability to walk, bite, chew and swallow.

The deletion encompassed 13 genes, among which 7 are indexed in OMIM database (http://www.omim.org/): *ABCC5*, *HTR3D, HTR3C*, *HTR3E, EIF2B5*, *DVL3*, *AP2M1*. Among these, *HTR3D, HTR3C, HTR3E* deserve special attention. Encoding subunits D, C and E of serotonin receptor type 3, these genes are expressed in various tissues, including the fetal brain. The role

of these subunits in receptor structure and function is not fully understood, but serotonin-mediated functions are known to be impaired in RTT. Altered serotonergic transmission was detected in prefrontal and motor cortices, along with engagement of hippocampus and cerebellum as the disease progress. This process is presumably involved in the pathophysiology of RTT motor phenotypes [4]. Therefore, the deletion indicates that serotonin receptor genes might be involved in RTT pathogenesis. Finally, deletions at this chromosomal region were never shown to cause RTT-like phenotype [5]. This study was supported by the Russian Science Foundation (project #14-35-00060).

References:

[1] Vorsanova SG, Iourov IY, Yurov YB. Neurological, genetic and epigenetic features of Rett syndrome. J Pediatr Neurol. 2004; 2(4):179-190.

[2] Vorsanova SG, Iurov IIu, Voinova VIu, Kurinnaia OS, Zelenova MA, Demidova IA, Ulas EV, Iurov IuB. [Subchromosomal microdeletion identified by molecular karyotyping using DNA microarrays (array CGH) in Rett syndrome girls negative for *MECP2* gene mutations]. Zh Nevrol Psikhiatr Im S S Korsakova. 2013; 113(10):63-8.

[3] Iourov IY, Vorsanova SG, Kurinnaia OS, Zelenova MA, Silvanovich AP, Yurov YB. Molecular karyotyping by array CGH in a Russian cohort of children with intellectual disability, autism, epilepsy and congenital anomalies. Mol Cytogenet. 2012; 5(1):46.

[4] De Filippis B, Chiodi V, Adriani W, Lacivita E, Mallozzi C, Leopoldo M, Domenici MR, Fuso A, Laviola G. Long-lasting beneficial effects of central serotonin receptor 7 stimulation in female mice modeling Rett syndrome. Front Behav Neurosci. 2015; 9:86.

[5] Dasouki M, Roberts J, Santiago A, Saadi I, Hovanes K. Confirmation and further delineation of the 3q26.33-3q27.2 microdeletion syndrome. Eur J Med Genet. 2014; 57(2-3):76-80.

11p15.5 duplications and Rett-like phenotype

Demidova I.A.[1,2,3], Vorsanova S.G.[1,2,3], Vasin K.S.[1,2,3], Zelenova M.A.[1,2,3], Kurinnaia O.S.[1,2,3], Shmitova N.S.[1,2,3], Yurov Y.B.[1,2,3], Iourov I.Y.[1,2,4]

[1]Research and Clinical Institute for Pediatrics named after Y.E. Veltishev at the Pirogov Russian National Research Medical University, Ministry of Health, Moscow, Russian Federation [2]Mental Health Research Center, Moscow, Russian Federation [3]Moscow State University of Psychology and Education, Moscow, Russian Federation [4]Department of Medical Genetics, Russian Medical Academy of Postgraduate Education, Ministry of Health, Moscow, Russian Federation.

Rett syndrome (RTT) is a severe neurodevelopmental disorder commonly associated with mutations in *MECP2*. However, several genes have been implicated into Rett syndrome like phenotype spectrum: *MECP2, CDKL5, FOXG1.* Lately, there has been a rapid growth in publications suggesting alterations to these genes causing RTT-like phenotypes [1]. However, associations between gross CNVs and this condition are still underappreciated. Nevertheless, a number of studies have indicated CNVs to be associated with RTT-like phenotypes [2]. This suggests CNV evaluation to be of basic and practical importance for RTT.

Here, using an original bioinformatics technology [3], we have analyzed microarray data on individuals with RTT-like phenotypes and intellectual disability (n=48). Among these individuals, two cases exhibited submicroscopic 11p15.5 duplications. These affected a genomic locus (chromosome 11p15.5; 171,667-747,717) spanning 17 genes indexed in OMIM (online Mendelian

inheritance in man), 5 of which are associated with a phonotypical outcome. For example, *HRAS* and *DRD4* are associated with congenital myopathy, Costello syndrome and autonomic nervous system dysfunction, attention deficit-hyperactivity disorder, respectively. Bioinformatic analysis has demonstrated *HRAS, MECP2, DRD4,* and *FOXG1* to share the interactome and Notch signaling pathway. It is to note that duplications within these chromosomal loci have never been associated with RTT-like phenotypes [4].

CNV analysis is able to reveal new mechanisms for genomic and monogenic diseases [5]. Here, a successful application of CNV analysis with bioinformatics has shown 11p15.5 duplications to cause RTT-like phenotype suggests this approach applicable for further studying RTT. This study was supported by the Russian Science Foundation (project #14-35-00060).

References:

[1] Gold WA, Christodoulou J. The utility of next-generation sequencing in gene discovery for mutation-negative patients with Rett syndrome. Front Cell Neurosci. 2015; 9:266.

[2] Lopes F, Barbosa M, Ameur A, Soares G, de Sá J, Dias AI, Oliveira G, Cabral P, Temudo T, Calado E, Cruz IF, Vieira JP, Oliveira R, Esteves S, Sauer S, Jonasson I, Syvänen AC, Gyllensten U, Pinto D, Maciel P. Identification of novel genetic causes of Rett syndrome-like phenotypes. J Med Genet. 2016; 53(3):190-9.

[3] Iurov IIu, Vorsanova SG, Saprina EA, Iurov IuB. [Identification of candidate genes of autism on the basis of molecular cytogenetic and in silico studies of the genome organization of chromosomal regions involved in unbalanced rearrangements]. Genetika. 2010; 46(10):1348-51.

[4] Baskin B, Choufani S, Chen YA, Shuman C, Parkinson N, Lemyre E, Micheil Innes A, Stavropoulos DJ, Ray PN, Weksberg R. High frequency of copy number variations (CNVs) in the chromosome 11p15 region in patients with Beckwith-Wiedemann syndrome. Hum Genet. 2014; 133(3):321-30.

[5] Iourov IY, Vorsanova SG, Yurov YB. Molecular cytogenetics and cytogenomics of brain diseases. Curr Genomics. 2008; 9(7):452-65.

Clinical and molecular characterization of X-linked mental retardation in Russian cohort of children with intellectual disability and congenital malformations

Voinova V.Y.[1,2,3], Vorsanova S.G.[1,2,3], Yurov Y.B.[1,2,3], Iourov I.Y.[1,2,4]

[1]Research and Clinical Institute for Pediatrics named after Y.E. Veltishev at the Pirogov Russian National Research Medical University, Ministry of Health, Moscow, Russian Federation [2]Mental Health Research Center, Moscow, Russian Federation [3]Moscow State University of Psychology and Education, Moscow, Russian Federation [4]Department of Medical Genetics, Russian Medical Academy of Postgraduate Education, Ministry of Health, Moscow, Russian Federation.

X-linked mental retardation (XLMR or X-linked intellectual disability/XLID) represents an umbrella term for hereditary diseases characterized by intellectual disability (significant limitations in cognitive abilities and social/behavioral adaptive skills) which are caused by X-linked mutations. The estimated cumulative prevalence of these diseases ranges from 1:1000 to 1.8:1000 [1, 2]. Addressing previously described cohort of children with intellectual disability and congenital malformations [3-5], XLMR/XLID was diagnosed in 6.54% of children with cognitive impairment, examined in a specialized genetic clinic. Specific quantitative phenotype scales were developed to quantify the disease severity in patients with Rett syndrome (RTT) and fragile X mental retardation syndrome (FRAXA). RTT was molecularly confirmed in 91% (315/354) among children selected by the quantitative phenotypic scale. FRAXA was confirmed in 40% (46/114) among selected cases. Premutations or full *FMR1* mutations were revealed in 84% (43/107) of relatives in FRAXA families emphasizing the

importance of family studies in this disease. Skewed X chromosome inactivation was detected in 45% (30/67) of girls with dominant X-linked diseases (RTT, Incontinentia Pigmenti type 2, Aicardi syndrome etc.), 20% (13/66) of their mothers and 46% (38/82) of asymptomatic women in XLMR/XLID boys families compared with 6.5% (5/76) of skewed X-inactivation cases in the control group of normal women with healthy children. X chromosome inactivation was skewed in 23% of females in families with male-specific mental retardation (non-confirmed XLNR/XLID). These data confirm skewed X-inactivation to feature heterozygous carriers of X-linked mutations. Accordingly, the phenotype outcomes in affected heterozygotes with X-linked dominant diseases depend on the degree and direction of the skewed X chromosome inactivation. Consequently, taking into account recent developments in molecular single-gene mutations (i.e. next-generation sequencing/NGS) [2, 6], a diagnostic algorithm, based on a stepwise application of clinical/genealogical analysis, X-linked NGS analysis and X chromosome inactivation, can be proposed. This study was supported by the Russian Science Foundation (project #14-15-00411).

References:

[1] des Portes V. X-linked mental deficiency. Handb Clin Neurol. 2013; 111:297-306.

[2] Tzschach A, Grasshoff U, Beck-Woedl S, Dufke C, Bauer C, Kehrer M, et al. Next-generation sequencing in X-linked intellectual disability. Eur J Hum Genet. 2015; 23(11):1513-8.

[3] Yurov YB, Vorsanova SG, Iourov IY, Demidova IA, Beresheva AK, Kravetz VS, Monakhov VV, Kolotii AD, Voinova-Ulas VY, Gorbachevskaya NL. Unexplained autism is frequently associated with low-level mosaic aneuploidy. J Med Genet. 2007; 44(8):521-5.

[4] Vorsanova SG, Iurov IIu, Kurinnaia OS, Voinova VIu, Iurov IuB. [Genomic abnormalities in children with mental retardation and autism: the use of comparative genomic

hybridization in situ (HRCGH) and molecular karyotyping with DNA-microchips (array CGH)]. Zh Nevrol Psikhiatr Im S S Korsakova. 2013; 113(8):46-9.
[5] Iourov IY, Vorsanova SG, Korostelev SA, Zelenova MA, Yurov YB. Long contiguous stretches of homozygosity spanning shortly the imprinted loci are associated with intellectual disability, autism and/or epilepsy. Mol Cytogenet. 2015; 8:77.
[6] Hu H, Haas SA, Chelly J, Van Esch H, Raynaud M, de Brouwer AP, et al. X-exome sequencing of 405 unresolved families identifies seven novel intellectual disability genes. Mol Psychiatry. 2016; 21(1):133-48.

Discovery of new genes in Rett syndrome patients by WES

Vidal Falcó S.[1], Lucariello M.[2], Vidal Ocabo E.[2], Brandi Tarrau N.M.[3], Gerotina Mora E.[1], Jurado P.[2], Esteller Badosa M. [2,4], Pineda Marfa M.[1], Armstrong Morón J.[3]

[1]Fundación Sant Joan de Déu, Barcelona, Spain. [2]Programa d'Epigenetica i Biologia del Cancer (PEBC), Institut d'Investigació Biomèdica de Bellvitge (IDIBELL), Barcelona, Spain. [3]Servicio de Genética Bioquímica&Rett, Hospital Universitari Sant Joan de Déu. Barcelona, Spain. [4]Departament de Ciencies Fisiològiques II, Facultat de Medicina, Universitat de Barcelona, Institució Catalana de Recerca i Estudis Avançats (ICREA), Barcelona, Spain.

Introduction: Rett syndrome (RTT) is a developmental disorder of early onset, genetic basis, dominant inheritance and X-linked. There are described three genes that cause RTT: MECP2, CDKL5 and FOXG1. However, the etiology of 15% of RTT patients still remains unknown. Thus, the aim of this project is to identify new candidate genes in a cohort of patients with RTT phenotype without genetic diagnosis by Whole Exome Sequencing (WES).

Material and Method: The patient and healthy parents without genetic diagnosis and negative CGHarray Cytoarray Plus (180K) (Agilent Microarrays) were analyzed by WES with TruSeq Sample Preparation Kit (Illumina). The filtering criteria

used were: search mutations with 1000g MAF below 0.05 in genes with dominant inheritance, de novo, X-linked, autosomal subject to imprinting and/or with functional impact in the CNS. For the validated mutations in genes related with gabaergic pathways (SLC6A1 and GABBR2), we performed RT-qPCR (TaqMan Gene Expression) and Western Blotting assay of RNA and protein extraction from peripheral blood.

Results: Most of the validated mutations are genes expressed in the central nervous system: ion channels and GABA/glutamate/acetylcholine pathways. The preliminary studies of the SLC6A1 and GABBR2 expression were not conclusive.

Conclusions: We do not only identify 1 gene which causes RTT-like phenotype. Pathway of genes has to be address to understand overlapping phenotype, instead to one disease only. Although blood tissue has convenient extraction, we cannot detect RNA and protein of these genes. Our next studies are performing RT-qPCR and Western Blotting assays with RNA and protein extraction from fibroblasts.

Genetic diagnosis of patients with overlapping clinic Rett-like by targeted panel of genes

Brandi Tarrau N.[1], Pacheco Fernández P.[1], Vidal Falcó S.[2], Blasco Pérez L.[2], García Cazorla A.[3], o'Callaghan M.[3], Ortez González C.I.[3], Poo Arguelles P.[3], Gean Molins E.[4], Pineda Marfa M.[2], García Peñas J.J.[5], Guitart M.[6], Armstrong Morón J.[1]

[1]Servicio de Genética Bioquímica&Rett [2]Fundación San Juan de Dios [3]Neurología y [4]Genética Clínica, Hospital Universitario San Juan de Dios, Barcelona [5]Neuropediatric Unit, Hospital Infantil Universitario Niño Jesús, Madrid. 6Corporación Sanitaria Parc Taulí, Barcelona.

Introduction: It has been studied patients with clinical Rett (RTT) without genetic diagnosis using the Next Generation Sequencing (NGS). This type of diseases requires clinical diagnosis. The finding of a mutation confirms the diagnosis, but not necessarily established it. NGS using targeted panel of genes facilitates the simultaneous study of causative genes of RTT and others whose mutation produces a similar or overlapping clinic, such Pitt Hopkins and Ohtahara syndromes.

Material and Methods: It has been designed a gene panel of 17 genes related to the clinical RTT-like presentation by HaloPlex Target technology. Enrichment System, for Illumina Sequencing. Sanger sequencing was used in exons not well covered. If do not find any change, MLPA was done by causative RTT genes.

Results: We have detected mutations in genes that do not cause RTT pathology in 14 of 187 studied patients with clinical Rett-like. A total of 8 patients presented mutations in STBX1 gene, related with Ohtahara syndrome and 6 patients were redirected as a Pitt-Hopkins, finding mutations in causative TCF4 gene. The database HGMD-professional, dbSNP, 1000 G and predictions pathology programs (Polyphen 2.0 and SIFT) were consulted. NGS variants have been verified by Sanger sequencing and studied the origin of the mutation in the parents.

Conclusion: The genetic study by NGS allows to study a larger number of genes associated with RTT simultaneously, redirecting genetic diagnosis to other syndromes. Significantly reduce response time and the cost of the study.

Improving genetic diagnosis using NGS: comparing WES vs panels of genes

Gerotina E.[1], Brandi N.M.[2], Vidal S.[1], O'Callaghan M.M.[3], Garcia-Cazorla A.[3], Pineda M.[1], Armstrong J.[2]

[1]FSJD. Barcelona [2]Molecular and Genetics Medicine Service [3]Neurology, HSJD. Barcelona

Abstract: Targeted-capture NGS strategies may be of choice for a first step molecular studies of intellectual disability and autism spectrum disorder in general and for Rett syndrome (RTT) in particular. Because the targeted region is smaller, gene panel strategies present an improvement in contrast to WES, as better sequence coverage can be achieved. For clinical diagnosis, this approach is highly valuable not only for detecting mutations, but also for being capable to rule out candidate genes as causative of the pathology, and consequently, a positive or negative diagnostic report for physicians and families may be done. Thus, the lack of false-negative results assures a major reliability, indispensable in the diagnostic field. Deeper coverage also favors the detection of copy number variants, which should also be ruled out in target genes, taking into account that NGS approaches do not display all kinds of genetic variation susceptible to cause a disease. Our experience in clinical and genetic diagnostic with RTT using targeted-NGS by panel of genes allow us to study a larger number of genes associated with RTT simultaneously, significantly reducing response time and the cost of the study. It also allows us to study other related clinical RTT

and thus to redirect the clinical diagnosis to another disease genes: Angelman syndrome, Pitt-Hopkins syndrome, Dravet syndrome,....Verification by Sanger sequencing of the progenitors of the mutations detected by NGS remains essential for their characterization as well as perform functional studies.

PROCEEDINGS S38

Spectrum of *MECP2* copy number variations in a Russian cohort of children with intellectual disability, congenital malformations, epilepsy and autism

Vorsanova S.G.[1,2,3], Yurov Y.B.[1,2,3], Voinova V.Y.[1,2,3], Kolotii A.D.[1,2], Demidova I.A.[1,2,3], Kurinnaia O.S.[1,2,3], Zelenova M.A.[1,2,3], Iourov I.Y.[1,2,4]

[1]Research and Clinical Institute for Pediatrics named after Y.E. Veltishev at the Pirogov Russian National Research Medical University, Ministry of Health, Moscow, Russian Federation [2]Mental Health Research Center, Moscow, Russian Federation [3]Moscow State University of Psychology and Education, Moscow, Russian Federation [4]Department of Medical Genetics, Russian Medical Academy of Postgraduate Education, Ministry of Health, Moscow, Russian Federation.

Microduplications of the long arm of the X chromosome involving *MECP2* are relatively common causes of neurodevelopmental disorders in males and, more rarely, females [1, 2]. In contrast to duplications, deletions of Xq28 involving *MECP2* cause milder classical and atypical Rett syndrome (RTT) [3]. Furthermore, mutations and copy number variations (CNV) of *MECP2* are significant contributors to the etiology of intellectual disability and neurobehavioral phenotypes [4, 5]. To determine the contribution of *MECP2* CNV to the etiology of intellectual disability,

congenital malformations, epilepsy and autism, we have analyzed corresponding cohort of children.

Using molecular karyotyping and original bioinformatics technology [3, 6, 7], 513 children with intellectual disability, congenital malformations, epilepsy and/or autism were studied. Duplications were detected in 4 males (0.8%) and corresponded to previous descriptions of Xq28 (*MECP2*) duplication syndrome [1, 7]. Deletions were detected in 12 females (2.3%) and corresponded to classical and atypical RTT [3]. Interestingly, the detection rates of *MECP2* CNV are similar to those detected previously in a variety of clinical cohorts [2, 3, 5, 7]. Finally, *MECP2* CNV (including both deletions and duplications) affects 3.1% of children in Russian cohort of children with intellectual disability, congenital malformations, epilepsy and autism. This suggests that *MECP2* CNV is the leading genetic cause of neurobehavioral phenotypes associated with single-gene CNV.

The study was supported by the Russian Science Foundation (project #14-15-00411).

References:

[1] Sanlaville D, Schluth-Bolard C, Turleau C. Distal Xq duplication and functional Xq disomy. Orphanet J Rare Dis. 2009; 4:4.

[2] Shimada S, Okamoto N, Ito M, Arai Y, Momosaki K, Togawa M, Maegaki Y, Sugawara M, Shimojima K, Osawa M, Yamamoto T. *MECP2* duplication syndrome in both genders. Brain Dev. 2013; 35(5):411-9.

[3] Iourov IY, Vorsanova SG, Voinova VY, Kurinnaia OS, Zelenova MA, Demidova IA, Yurov YB. Xq28 (*MECP2*) microdeletions are common in mutation-negative females with Rett syndrome and cause mild subtypes of the disease. Mol Cytogenet. 2013; 6(1):53.

[4] Iurov IIu, Vorsanova SG, Iurov IuB. [Nervous and mental diseases in boys and mutations in *MECP2* gene]. Zh Nevrol Psikhiatr Im S S Korsakova. 2004; 104(10):73-80.

[5] Lugtenberg D, Kleefstra T, Oudakker AR, Nillesen WM, Yntema HG, Tzschach A, et al. Structural variation in Xq28: *MECP2* duplications in 1% of patients with unexplained XLMR and in 2% of male patients with severe encephalopathy. Eur J Hum Genet. 2009; 17(4):444-53.

[6] Iourov IY, Vorsanova SG, Korostelev SA, Zelenova MA, Yurov YB. Long contiguous stretches of homozygosity spanning shortly the imprinted loci are associated with intellectual disability, autism and/or epilepsy. Mol Cytogenet. 2015; 8:77.

[7] Voinova VY, Vorsanova SG, Yurov YB, Kolotiy AD, Davidova YI, Demidova IA, Novikov PV, Iourov IY. [Clinical and genetic characteristics of the X chromosome distal long arm microduplications encompassing the *MECP2* gene]. Zh Nevrol Psikhiatr Im S S Korsakova. 2015; 115(10):10-6.

Mosaic 1p36.22 duplication and Rett-like phenotype

Zelenova M.A.[1,2,3], Vorsanova S.G.[1,2,3], Yurov Y.B.[1,2,3], Kurinnaia O.S.[1,2,3], Iourov I.Y.[1,2,4]

[1]Mental Health Research Center, Moscow, Russian Federation [2]Research and Clinical Institute for Pediatrics named after Y.E. Veltishev at the Pirogov Russian National Research Medical University, Ministry of Health, Moscow, Russian Federation [3]Moscow State University of Psychology and Education, Moscow, Russian Federation [4]Department of Medical Genetics, Russian Medical Academy of Postgraduate Education, Ministry of Health, Moscow, Russian Federation.

Structural genomic rearrangements and unbalanced chromosome abnormalities at 1p36 are associated microdeletion and microduplication syndromes as well as a variety of clinically heterogeneous outcomes [1-4]. Regardless of heterogeneous phenotypic consequences, duplications at 1p36 were never associated with Rett syndrome or Rett-like phenotypes [5, 6]. Molecular karyotyping (array CGH platform: NimblGen 135K) and an original bioinformatic technique [5] allowed us the detection of a mosaic 1p36.22 duplication affecting 11,244,644-12,399,379 genomic region in a girl with Rett-like clinical manifestations. Thirty two genes were duplicated, 19 of which are indexed in OMIM (online Mendelian inheritance in man). Bioinformatic analysis has highlighted the following genes as those likely contributing to Rett-

like phenotype: *MTHFR* (associated with neural tube defects and susceptibility to schizophrenia) and *MFN2* (associated with Charcot-Marie-Tooth disease and hereditary motor and sensory neuropathy VI). Interestingly, mosaic structural chromosomal rearrangements are known to produce local genomic instability throughout the ontogeny creating risks of additional phenotypic manifestations due to further genome rearrangements [7, 8]. Consequently, a molecular cytogenetic monitoring [7] is warranted in this case. To this end, our data provide the first evidence that mosaic duplications at 1p36 can underlie Rett-like phenotypes. The study was supported by the Russian Science Foundation (project #14-35-00060).

References:

[1] Battaglia A. Del 1p36 syndrome: a newly emerging clinical entity. Brain Dev. 2005; 27(5):358-61.

[2] Polityko A, Starke H, Rumyantseva N, Claussen U, Liehr T, Raskin S. Three cases with rare interstitial rearrangements of chromosome 1 characterized by multicolor banding. Cytogenet Genome Res. 2005; 111(2):171-4.

[3] Iourov IY, Vorsanova SG, Kirillova EA, Yurov YB. First case of del(1)(p36.2p33) in a fetus delivered stillborn. Prenat Diagn. 2006; 26(11):1092-3.

[4] Jordan VK, Zaveri HP, Scott DA. 1p36 deletion syndrome: an update. Appl Clin Genet. 2015; 8:189-200.

[5] Vorsanova SG, Iurov IIu, Voinova VIu, Kurinnaia OS, Zelenova MA, Demidova IA, Ulas EV, Iurov IuB. [Subchromosomal microdeletion identified by molecular karyotyping using DNA microarrays (array CGH) in Rett syndrome girls negative for *MECP2* gene mutations]. Zh Nevrol Psikhiatr Im S S Korsakova. 2013; 113(10):63-8.

[6] Lopes F, Barbosa M, Ameur A, Soares G, de Sá J, Dias AI, Oliveira G, Cabral P, Temudo T, Calado E, Cruz IF, Vieira JP, Oliveira R, Esteves S, Sauer S, Jonasson I, Syvänen AC, Gyllensten U, Pinto D, Maciel P. Identification of novel genetic causes of Rett syndrome-like phenotypes. J Med Genet. 2016; 53(3):190-9.

[7] Iourov IY, Vorsanova SG, Liehr T, Monakhov VV, Soloviev IV, Yurov YB. Dynamic mosaicism manifesting as loss, gain and rearrangement of an isodicentric Y chromosome in a male child with growth retardation and abnormal external genitalia.

Cytogenet Genome Res. 2008; 121(3-4):302-6.
[8] Yurov YB, Vorsanova SG, Iourov IY. Ontogenetic variation of the human genome. Curr Genomics. 2010; 11(6):420-5.

PROCEEDINGS S40

Rett-like phenotype due to a 14q11.2 microdeletion

Kurinnaia O.S.[1,2,3], Vorsanova S.G.[1,2,3], Yurov Y.B.[1,2,3], Iourov I.Y.[1,2,4]

[1]Research and Clinical Institute for Pediatrics named after Y.E. Veltishev at the Pirogov Russian National Research Medical University, Ministry of Health, Moscow, Russian Federation [2]Mental Health Research Center, Moscow, Russian Federation [3]Moscow State University of Psychology and Education, Moscow, Russian Federation [4]Department of Medical Genetics, Russian Medical Academy of Postgraduate Education, Ministry of Health, Moscow, Russian Federation.

We describe a 6 year old girl with a Rett-like phenotype who was studied by molecular karyotyping (NimblGen 135K platform) and a bioinformatic technique as described earlier [1-3]. We were able to detect a microdeletion at 14q11.2 (size: approximately 819.5 kb) spanning 40 genes, among which 19 are indexed in OMIM (online Mendelian inheritance in man): *EDDM3A*, *EDDM3B*, *RNASE6*, *NASE1*, *RNASE3*, *RNASE2*, *SLC39A2*, *RNASE7*, *NDRG2*, *RNASE8*, *ARHGEF40*, *ZNF219*, *HNRNPC*, *RPGRIP1*, *SUPT16H*, *CHD8*, *RAB2B*, *TOX4*, *METTL3*. Bioinformatics analysis suggested *CHD8*, *ZNF219*, and *SUPT16H* as candidate genes for the Rett-like phenotype. According to the available literature, 14q11.2 microdeletions were not primarily associated with Rett-like phenotype [3, 4]. Interestingly, these microdeletions commonly involve *CHD8*, which is associated with autistic spectrum disorders [5, 6]. Additionally, we have retrospectively analyzed a cohort of 400 patients with speech and developmental delay, intellectual disability and/or autism. In 3

patients, copy number variations affected 14q11.2. One patient had speech and development delay, visceromegaly, tall stature, hypoplastic thymus, hydrocephaly, secondary immunodeficiency, round face, large low-set ears and demonstrated microduplication at this chromosomal locus. Another patient (a girl with development delay, intellectual disability, and convergent strabismus) exhibited a triplication at 14q11.2. Finally, a girl with developmental delay, protruding forehead, clinodactyly, palpebral fissures, midface hypoplasia, low-set dysplastic auricles, myopia and muscular hypotonia was found to have a mosaic deletion at 14q11.2. According to genotype-phenotype correlations, we have speculated that the index case of 14q11.2 deletion is associated with Rett-like phenotype. To date, such chromosome imbalances have not been ever detected in children with Rett-like features. The study was supported by the Russian Science Foundation (project #14-35-00060).

References:

[1] Iourov IY, Vorsanova SG, Voinova VY, Kurinnaia OS, Zelenova MA, Demidova IA, Yurov YB. Xq28 (MECP2) microdeletions are common in mutation-negative females with Rett syndrome and cause mild subtypes of the disease. Mol Cytogenet. 2013; 6(1):53.

[2] Iourov IY, Vorsanova SG, Kurinnaia OS, Yurov YB. An interstitial 20q11.21 microdeletion causing mild intellectual disability and facial dysmorphisms. Case Rep Genet. 2013; 2013:353028.

[3] Vorsanova SG, Iurov IIu, Voinova VIu, Kurinnaia OS, Zelenova MA, Demidova IA, Ulas EV, Iurov IuB. [Subchromosomal microdeletion identified by molecular karyotyping using DNA microarrays (array CGH) in Rett syndrome girls negative for *MECP2* gene mutations]. Zh Nevrol Psikhiatr Im S S Korsakova. 2013; 113(10):63-8.

[4] Lopes F, Barbosa M, Ameur A, Soares G, de Sá J, Dias AI, Oliveira G, Cabral P, Temudo T, Calado E, Cruz IF, Vieira JP, Oliveira R, Esteves S, Sauer S, Jonasson I, Syvänen AC, Gyllensten U, Pinto D, Maciel P. Identification of novel genetic causes of Rett syndrome-like phenotypes. J Med Genet. 2016; 53(3):190-9.

[5] Zahir F, Firth HV, Baross A, Delaney AD, Eydoux P, Gibson WT, Langlois S, Martin H, Willatt L, Marra MA,

Friedman JM. Novel deletions of 14q11.2 associated with developmental delay, cognitive impairment and similar minor anomalies in three children. J Med Genet. 2007; 44(9):556-61.
[6] Prontera P, Ottaviani V, Toccaceli D, Rogaia D, Ardisia C, Romani R, Stangoni G, Pierini A, Donti E. Recurrent ~100 Kb microdeletion in the chromosomal region 14q11.2, involving CHD8 gene, is associated with autism and macrocephaly. Am J Med Genet A. 2014; 164A(12):3137-41.

PROCEEDINGS S41

Rett syndrome variome

Vorsanova S.G.[1,2,3], Yurov Y.B.[1,2,3], Zelenova M.A.[1,2,3], Kurinnaia O.S.[1,2,3], Vasin K.S.[1,2,3], Shmitova N.S.[2], Ratnikov A.M.[2], Voinova V.Y.[1,2,3], Iourov I.Y.[1,2,4]

[1]Research and Clinical Institute for Pediatrics named after Y.E. Veltishev at the Pirogov Russian National Research Medical University, Ministry of Health, Moscow, Russian Federation [2]Mental Health Research Center, Moscow, Russian Federation [3]Moscow State University of Psychology and Education, Moscow, Russian Federation [4]Department of Medical Genetics, Russian Medical Academy of Postgraduate Education, Ministry of Health, Moscow, Russian Federation.

Surveying the human variome (all the structural variants in the genome) provides a way to determine genomic disease mechanisms through the discovery of pathogenic genetic changes. Recently, it has been shown that variome analysis is able to identify genetic defects underlying a certain phenotype both in a case-by-case manner and in a set of individuals suffering from similar pathology [1-3]. Although Rett syndrome (RTT) is considered a monogenic disease resulting from *MECP2* mutations, there is convincing evidence that a proportion of RTT cases are likely to be caused by mutations/copy number variations (CNV) in other genes or genomic loci [4, 5].

Here, using molecular karyotyping and original bioinformatic technology (as described earlier

[5, 6]), we have studied the RTT variome in children without detectable *MECP2* mutations. In addition to deletions of Xq28 (*MECP2*), we were able to identify a number of genomic regions that are deleted and/or duplicated in RTT: 1p36.22, 1q21, 2q12.3q13, 2q13, 3p13, 3q27.1, 11p15.5, 11p14.3, 11p13, 14q11.2, 15q11.2, 15q14, 22q11.21, Xp22.13. *In silico* analysis by original bioinformatics workflow (genome-epigenome-interactome-metabolome) has allowed us to identify candidate genes for RTT or RTT-like phenotype: *MTHFR* and *MFN2* (1p36.22), *GJA5* and *GJA8* (1q21), *MALL* (2q12.3q13), *FBLN7* and *MERTK* (2q13), *FOXP1* (3p13), *ABCC5*, *EIF2B5*, *DVL3*, *AP2M1* (3q27.1), *HRAS* (11p15.5), *FANCF* (11p14.3), *CD44* and *SLC1A2* (11p13), *CHD8* (14q11.2), *UBE3A* (15q11.2), *GJD2* and *ACTC1* (15q14), *PI4KA* (22q11.21), *CDKL5* (Xp22.13/previously associated with atypical RTT). All these genes are either associated with neurological pathology resembling RTT or are involved in key processes of regulating central nervous system development and functioning. A number of these genes demonstrate brain-specific expression at different ontogenetic periods. Extensive interactomic analysis has demonstrated that these genes share interactome and pathways with “core” RTT genes (i.e. *MECP2, CDKL5,* and *FOXG1*). Consequently, we have come to a conclusion that a kind of RTT disease pathway appears to exist.

High-resolution genome analysis in combination with bioinformatics analysis of functional consequences of genomic changes allows the prediction of disease risk and preventing of disease [7]. To determine the genetic

mechanisms, the knowledge of all the (potentially) pathogenic genomic variations is mandatory. Moreover, this knowledge is able to indicate a diseases pathway [1-3, 6, 7]. Regardless of monogenic nature of RTT, variome analysis seems to be effective for uncovering disease mechanisms in *MECP2*-mutation negative cases. Therefore, one can suggest that this approach is able to identify new genetic causes of RTT-like phenotypes and related diseases. This study was supported by the Russian Science Foundation (project #14-15-00411).

References:

[1] Choy M, Movassagh M, Foo R. The human variome: genomic and epigenomic diversity. EMBO Mol Med. 2011; 3(10):573-4.

[2] Lopes P, Dalgleish R, Oliveira JL. WAVe: web analysis of the variome. Hum Mutat. 2011; 32(7):729-34.

[3] Iourov IY, Vorsanova SG, Yurov YB. Somatic cell genomics of brain disorders: a new opportunity to clarify genetic-environmental interactions. Cytogenet Genome Res. 2013; 139(3):181-8.

[4] Vorsanova SG, Iourov IY, Yurov YB. Neurological, genetic and epigenetic features of Rett syndrome. J Pediatr Neurol. 2004; 2(4):179-190.

[5] Vorsanova SG, Iurov IIu, Voinova VIu, Kurinnaia OS, Zelenova MA, Demidova IA, Ulas EV, Iurov IuB. [Subchromosomal microdeletion identified by molecular karyotyping using DNA microarrays (array CGH) in Rett syndrome girls negative for *MECP2* gene mutations]. Zh Nevrol Psikhiatr Im S S Korsakova. 2013; 113(10):63-8.

[6] Iourov IY, Vorsanova SG, Liehr T, Kolotii AD, Yurov YB. Increased chromosome instability dramatically disrupts neural genome integrity and mediates cerebellar degeneration in the ataxia-telangiectasia brain. Hum Mol Genet. 2009; 18(14):2656-69.

[7] Bromberg Y. Building a genome analysis pipeline to predict disease risk and prevent disease. J Mol Biol. 2013; 425(21):3993-4005

.

X-linked intellectual disability due to submicroscopic unbalanced rearrangements of chromosome X

Iourov I.Y.[1,2,3], Vorsanova S.G.[1,2,4], Zelenova M.A.[1,2,4], Kurinnaia O.S.[1,2,4], Vasin K.S.[1,2,4], Ratnikov A.M.[1], Yurov Y.B.[1,2,4]

[1]Mental Health Research Center, Moscow, Russian Federation [2]Research and Clinical Institute for Pediatrics named after Y.E. Veltishev at the Pirogov Russian National Research Medical University, Ministry of Health, Moscow, Russian Federation [3]Moscow State University of Psychology and Education, Moscow, Russian Federation [4]Department of Medical Genetics, Russian Medical Academy of Postgraduate Education, Ministry of Health, Moscow, Russian Federation.

X-linked intellectual disability (XLID) is commonly associated with chromosome X rearrangements and copy number variations (CNV) encompassing X-linked genes [1, 2]. However, high-resolution microarray analyses of XLID-associated chromosome rearrangements are rarely described in the available literature. Here, we have studied 312 patients with intellectual disability, autism and congenital malformations using molecular karyotyping (resolution: more than 1 kb) and original bioinformatic technology for assessing pathogenic values of CNV [3, 4].

XLID-associated genomic rearrangements were revealed in 11 children (3.5%). These were mosaic deletion at Xp22.32p22.2 (4,700 kb/female) with a deletion at Xp22.31 (710 kb), deletion at Xp22.12 (1,467 kb/female), duplication at Xp22.31 (616 kb/female), duplication at Xp21.2 (254 kb/male), mosaic deletion at Xq21.2q21.33 (8,800 kb/female), deletion at Xq21.31 (1200 kb/male), mosaic deletion at Xq26.2q26.3 (3,300 kb/female), mosaic deletion at Xq28 (1,600 kb/female). Three children had

multiple X chromosome rearrangements. The sex ratio was 7/4 (girls/boys).

It is to note that X-linked pathology is able to explain the male preponderance in the prevalence of neurobehavioral diseases (i.e. intellectual disability and autism) [5]. Here, we were able to show that XLID-associated chromosome rearrangements are also common in female patients. Furthermore, taking onto account the estimated prevalence of asymptomatic carriers of X-linked mutations and/or X chromosome rearrangements (CNVs) [6, 7], one can further speculate about the contribution of the chromosome/genome pathology to human morbidity. Mosaic chromosome rearrangements were common in this cohort. Taking into account that somatic chromosomal mosaicism has been recognized as an important, yet still difficult to determine, contributor to the etiology of intellectual disability [8]. Our study reveals new twists on the role of submicroscopic unbalanced rearrangements of chromosome X in XLID suggesting that these chromosome rearrangements are more important contributors to human morbidity than previously recognized. The study was supported by the Russian Science Foundation (project #14-15-00411).

References:

[1] Isrie M, Froyen G, Devriendt K, de Ravel T, Fryns JP, Vermeesch JR, Van Esch H. Sporadic male patients with intellectual disability: contribution of X-chromosome copy number variants. Eur J Med Genet. 2012; 55(11):577-85.

[2] Mignon-Ravix C, Cacciagli P, Choucair N, Popovici C, Missirian C, Milh M, et al. Intragenic rearrangements in X-linked intellectual deficiency: results of a-CGH in a series of 54 patients and identification of TRPC5 and KLHL15 as potential XLID genes. Am J Med Genet A. 2014; 164A(8):1991-7.

[3] Iourov IY, Vorsanova SG, Yurov YB. In silico molecular cytogenetics: a bioinformatic approach to prioritization of candidate genes and copy number variations for basic

and clinical genome research. Mol Cytogenet. 2014; 7(1):98.

[4] Iourov IY, Vorsanova SG, Zelenova MA, Korostelev SA, Yurov YB. Genomic copy number variation affecting genes involved in the cell cycle pathway: implications for somatic mosaicism. Int J Genomics. 2015; 2015:757680.

[5] Iourov IY, Yurov YB, Vorsanova SG. Mosaic X chromosome aneuploidy can help to explain the male-to-female ratio in autism. Med Hypotheses. 2008; 70(2):456.

[6] Iurov IIu, Willard L, Vorsanova SG, Demidova IA, Goĭko EA, Shal'nova SA, Shkol'nikova MA, Olfer'ev AM, Iurov IuB. [X chromosome inactivation pattern in elderly women over 70 years of age]. Tsitol Genet. 2004; 38(4):49-54.

[7] Fieremans N, Van Esch H, Holvoet M, Van Goethem G, Devriendt K, Rosello M, Mayo S, et al. Identification of intellectual disability genes in female patients with a skewed X inactivation pattern. Hum Mutat. 2016; doi: 10.1002/humu.23012.

[8] Iourov IY, Vorsanova SG, Yurov YB. Chromosomal mosaicism goes global. Mol Cytogenet. 2008; 1:26.

PROCEEDINGS S43

X-linked intellectual disability related genes disrupted by balanced X-autosome translocations

Moysés-Oliveira M.[1], Santos Guilherme R.[1,2], Meloni V.A.[1], Di Battista A.[1], Berlim de Mello C.[3], Bragagnolo S.[1], Moretti-Ferreira D.[4], Kosyakova N.[2], Liehr T.[2], Carvalheira G.M.[1], Melaragno M.I.[1]

[1]Department of Morphology and Genetics, Genetics Division, Universidade Federal de S~ao Paulo, Sao Paulo, Brazil [2]Jena University Hospital, Friedrich Schiller University, Institute of Human Genetics, Jena, Germany [3]Department of Psychobiology, Universidade Federal de S~ao Paulo, Sao Paulo, Brazil [4]Departament of Genetics, Instituto de Biocincias de Botucatu, Universidade Estadual de S~ao Paulo, Sao Paulo, Brazil

Chromosomal rearrangements involving X-chromosome have been studied for identification of X-linked intellectual disability-causing genes. Here a fine-mapping of the breakpoints in four women with balanced Xautosome translocations and variable phenotypes was done; the goal was to investigate the corresponding genetic contribution to intellectual

disability. Impact of the gene interruptions in transcription and the consequences of their functional impairment in neurodevelopment are discussed. Three patients presented with cognitive impairment, reinforcing the association between the disrupted genes (TSPAN7—MRX58, KIAA2022—MRX98, and IL1RAPL1—MRX21/34) and intellectual disability. While no expression of TSPAN7 and KIAA2022 in the patients was seen, the unexpected expression of IL1RAPL1 suggested a fusion transcript ZNF611-IL1RAPL1 under the control of the ZNF611 promoter gene disrupted at the autosomal breakpoint. The X-chromosomal breakpoint definition in the fourth patient, a woman with normal intellectual abilities, revealed disruption of the ZDHHC15 gene (MRX91). The expression assays did not detect ZDHHC15 gene expression in the patient, thus questioning its involvement in intellectual disability. Overall, revealing the disruption of an X-linked intellectual disability-related gene in patients with balanced X-autosome translocation is a useful tool for a better characterization of critical genes in neurodevelopment. This study was supported by Fundacao de Amparoa Pesquisa do Estado de Sao Paulo (FAPESP # 2011/51690-1).

3. Rare diseases

20p12.3 deletion is rare cause of syndromic cleft palate: case report and review of literature

Amasdl S.[1,2], Natiq A.[2,3], Sbiti A.[2], Zerkaoui M.[1,2], Lyahyai J.[1], Amzazi S.[3], Liehr T.[4], Sefiani A.[1,2]

[1]Centre de Génomique Humaine, Faculté de Médecine et de Pharmacie, Université Mohammed V, Rabat, Morocco [2]Département de Génétique Médicale, Institut National d'Hygiène, Rabat, Morocco [3]Faculté des Sciences, Université Mohammed V, Rabat, Morocco [4]Institut de Génétique Humaine, Hôpital Universitaire de Jena, Jena, Germany.

Orofacial cleft (OFC) is one of the most common congenital malformations with a global incidence of approximately 1/700 live births. Clinically, OFCs can be syndromic or non-syndromic. A 5 years old boy admitted for genetic evaluation because of psychomotor delay, failure to thrive, dysmorphic features and cleft palate. Banding cytogenetics showed a notably short p arm of one chromosome20. Molecular cytogenetic analysis identified the derivative chromosome 20 as a de novo 20p12.3 deletion. Overall, we present in this paper a Moroccan patient with syndromic cleft palate caused by a de novo 20p12.3 deletion, and we highlight the interest of molecular cytogenetics in the diagnosis confirmation of chromosomal rearrangement. In practice, 20p12.3 deletion should be considered as an etiological diagnosis in the case of syndromic cleft palate.

22q11.21 duplication detected by array CGH and confirmed by FISH: a case report

Kolotii A.D.[1,2], Vorsanova S.G.[1,2,3], Voinova V.Y.[1,2,3], Yurov Y.B.[1,2,3], Iourov I.Y.[1,2,4]

[1]Research and Clinical Institute for Pediatrics named after Y.E. Veltishev at the Pirogov Russian National Research Medical University, Ministry of Health, Moscow, Russian Federation [2]Mental Health Research Center, Moscow, Russian Federation [3]Moscow State University of Psychology and Education, Moscow, Russian Federation [4]Department of Medical Genetics, Russian Medical Academy of Postgraduate Education, Ministry of Health, Moscow, Russian Federation.

22q11.2 duplication syndrome (OMIM 608363) results from recurrent 1.5-3 Mb proximal tandem duplication. The 22q11.21 duplication phenotype appears to be rather mild and highly variable. Duplication 22q11.2 occurs much less frequently as to deletions at this chromosomal region (DiGeorge or velocardiofacial syndromes — DGS/VCFS). Most individuals with 22q11.21 duplication have inherited duplication from an asymptomatic parent. The duplication is undetectable by G-banded karyotyping. The commonest findings in symptomatic individuals with 22q11.2 duplication are intellectual disability, psychomotor development delay, growth retardation, and muscular hypotonia [1].

We present a 1 year old boy with presumably normal karyotype (karyotyping at 550 band resolution) born at 38 weeks (weight – 2300 g; length – 50 cm) with esophageal atresia and hypospadias. Heart defects (ventricular septal defect, patent foramen ovale, the open aortic flow) were noted. Significant

motor development delay, short stature, microcephaly, epicanthic folds, broad nasal bridge, a short nose with a thick tip, long filtrum, thin upper lip, high narrow palate, abnormal growth of teeth, short neck, sparse hair, hypertelorism nipples, limb shortening, transverse palmar crease, brachydactyly, small hands and feet, overlapping toes, expansion of the lateral ventricles and the brain ventricle III were noted, as well. Parents are not consanguineous.

Array CGH performed as previously described [2] revealed a 22q11.21 duplication (~2.5Mb) (18972449-21462567) spanning 37 genes (*DGCR2*, *DGCR14*, *TSSK2*, *GSC2*, *SLC25A1*, *CLTCL1*, *HIRA*, *MRPL40*, *UFD1L*, *CDC45*, *CLDN5*, *SEPT5*, *GP1BB*, *TBX1*, *GNB1L*, *TXNRD2*, *COMT*, *ARVCF*, *DGCR8*, *TRMT2A*, *RANBP1*, *ZDHHC8*, *RTN4R*, *DGCR6L*, *RIMBP3*, *ZNF74*, *SCARF2*, *MED15*, *PI4KA*, *SERPIND1*, *SNAP29*, *CRKL*, *LZTR1*, *THAP7*, *P2RX6*, *SLC7A4*, *BCRP2*). Duplication was confirmed by fluorescence *in situ* hybridization, showing signal amplification in metaphase spreads and signal doubling in interphase nuclei [3]. The clinical variability and lack of some symptoms in a large proportion of 22q11.21 duplication carriers suggests the involvement of epigenetic factors. Possiblly, increased expression of TBX1 combined with dysregulation of other genes is discussed in the literature [4]. The study was supported by the Russian Science Foundation (project #14-35-00060).

References:

[1] Wentzel C, Fernström M, Ohrner Y, Annerén G, Thuresson AC. Clinical variability of the 22q11.2 duplication syndrome. Eur J Med Genet. 2008; 51(6):501-10.

[2] Iourov IY, Vorsanova SG, Voinova VY, Kurinnaia OS, Zelenova MA, Demidova IA, Yurov YB. Xq28 (MECP2) microdeletions are common in mutation-negative females with Rett syndrome and

cause mild subtypes of the disease. Mol Cytogenet. 2013; 6(1):53.

[3] Yurov YB, Vorsanova SG, Liehr T, Kolotii AD, Iourov IY. X chromosome aneuploidy in the Alzheimer's disease brain. Mol Cytogenet. 2014; 7(1):20.

[4] Weisfeld-Adams JD, Edelmann L, Gadi IK, Mehta L. Phenotypic heterogeneity in a family with a small atypical microduplication of chromosome 22q11.2 involving TBX1. Eur J Med Genet. 2012; 55(12):732-6.

PROCEEDINGS S46

Association of new deletion/duplication region at chromosome 1p21 with intellectual disability, severe speech deficit and autism spectrum disorderlike behavior: an all-in approach to solving the DPYD enigma

Brečević L.[1,2], Rinčić M.[1,2,3], Liehr T.[3], Galić I.[4], Borovečki F.[2]

[1]Croatian Institute for Brain Research, University of Zagreb Medical School, Šalata 12, 10000 Zagreb, Croatia [2]Department for Functional Genomics, Center for Translational and Clinical Research, University of Zagreb Medical School, University Hospital Center Zagreb, Šalata 2, 10000 Zagreb, Croatia [3]Jena University Hospital, Friedrich Schiller University, Institute of Human Genetics, Kollegiengasse 10, 07743 Jena, Germany [4]Center for Rehabilitation Stančić, Stančić bb, 10370 Stančić, Croatia

A yet unreported neocentric small supernumerary marker chromosome (sSMC) derived from chromosome 1p21.3p21.2 was found in 80% of the lymphocytes in a male patient with intellectual disability, severe speech deficit, mild dysmorphic features, and hyperactivity with elements of autism spectrum disorder (ASD). Several important neurodevelopmental genes were affected by the 3.56 Mb copy number gain of 1p21.3p21.2, which may be considered reciprocal in gene content to the recently recognized 1p21.3 microdeletion syndrome. Both 1p21.3 deletions and the presented duplication display

overlapping symptoms, fitting the same disorder category. Contribution of coding and non-coding genes to the phenotype is discussed in the light of cellular and intercellular homeostasis disequilibrium.Although miR-137 appears to be the major player in the 1p21.3p21.2 region, deregulation of the DPYD (dihydropyrimidine dehydrogenase) gene may potentially affect neighboring genes underlying the overlapping symptoms present in both the copy number loss and copy number gain of 1p21. Namely, the all-in approach revealed that DPYD is a complex gene whose expression is epigenetically regulated by long non-coding RNAs (lncRNAs) within the locus. Furthermore, the long interspersed nuclear element-1 (LINE-1) L1MC1 transposon inserted in DPYD intronic transcript 1 (DPYD-IT1) lncRNA with its parasites, TcMAR-Tigger5b and pair of Alu repeats appears to be the "weakest link" within the DPYD gene liable to break. Identification of the precise mechanism through which DPYD is epigenetically regulated, and underlying reasons why exactly the break (FRA1E) happens, will consequently pave the way toward preventing severe toxicity to the antineoplastic drug 5-fluorouracil (5-FU) and development of the causative therapy for the dihydropyrimidine dehydrogenase deficiency.

An interstitial deletion of chromosome 11 in a 2-year-old boy with developmental delay and microanomalies

Kravets V.S.[1,2,3], Vorsanova S.G.[1,2,3], Kolotii A.D.[1,2], Dantsev I.S.[1], Yurov Y.B.[1,2,3], Iourov I.Y.[1,2,4]

[1]Research and Clinical Institute for Pediatrics named after Y.E. Veltishev at the Pirogov Russian National Research Medical University, Ministry of Health, Moscow, Russian Federation [2]Mental Health Research Center, Moscow, Russian Federation [3]Moscow State University of Psychology and Education, Moscow, Russian Federation [4]Department of Medical Genetics, Russian Medical Academy of Postgraduate Education, Ministry of Health, Moscow, Russian Federation.

Different chromosomal anomalies are a common reason of rare diseases presenting with developmental delays and congenital malformations. Among those, chromosomal deletions take place rather frequently and have more severe consequences in comparison with translocations, duplications, ring chromosomes, etc. Thorough characterization of these chromosomal rearrangements helps to define genes involved in a rearrangement, to give prognosis, and to perform genetic counseling. We describe a 2-year-old boy with developmental delay, hypertelorism, upslanted palpebral fissures, strabismus, epicantus, flat nasal bridge, high broad forehead, low-set ears, micrognatia, short neck, camptodactyly II-IV and some features of Moebius syndrome. Blood culture, differential staining and cytogenetic analysis were performed as described elsewhere [1]. The karyotype of the proband was 46,XY, del(11)(q2?1q2?3). It should be mentioned that the boy was previously karyotyped in his native city, but that analysis was not performed proper enough to suggest the breakpoints, and the deletion was first thought to be

terminal (46,XY,del(11)(q22)), not interstitial, as we established later. We recommended performing molecular karyotyping to define more precisely the deletion breakpoints, which is to be performed in nearest future. Karyotyping of the boy's parents was recommended for correct genetic counseling of the family, as well.

It is to note that interstitial 11q deletions are extremely rare [2]. Moreover, chromosome regions suggested being involved in the index case are also known to be prone to rearrange at the chromosomal level [3]. At this stage, one has to conclude that modern molecular cytogenetic methods based on *in situ* hybridization, such as array CGH, should be performed in all the cases of complex or cryptic chromosomal rearrangements. It helps to characterize the rearrangement precisely, define genes involved in the deletion and find new rare chromosomal diseases (syndromes). *This study was supported by the Russian Science Foundation (project #14-15-00411).*

References:

[1] Vorsanova SG, Iourov IY, Beresheva AK, Demidova IA, Monakhov VV, Kravets VS, Bartseva OB, Goyko EA, Soloviev IV, Yurov YB. Non-disjunction of chromosome 21, alphoid DNA variation, and sociogenetic features of Down syndrome. Tsitol Genet. 2005; 39(6):30-6.

[2] Nacinovich R, Villa N, Redaelli S, Broggi F, Bomba M, Stoppa P, et al. Interstitial 11q deletion: genomic characterization and neuropsychiatric follow up from early infancy to adolescence and literature review. BMC Res Notes. 2014; 7:248.

[3] Du N, Baker PM, Do TU, Bien C, Bier-Laning CM, Singh S, Shih SJ, Diaz MO, Vaughan AT. 11q21.1-11q23.3 is a site of intrinsic genomic instability triggered by irradiation. Genes Chromosomes Cancer. 2010; 49(9):831-43.

Complex intrachromosomal rearrangement in 1q leading to 1q32.2 microdeletion: a potential role of *SRGAP2* in the gyrification of cerebral cortex

Rincic M.[1], Rados M.[1], Krsnik Z.[1], Gotovac K.[2], Borovecki F.[2, 3], Liehr T.[4], Brecevic L.[1]

[1]Croatian Institute for Brain Research, School of Medicine University of Zagreb, Salata 12, 10000 Zagreb, Croatia [2]Department for Functional Genomics, Center for Translational and Clinical Research, University of Zagreb School of Medicine, and University Hospital Center Zagreb, Šalata 2, 10 000 Zagreb, Croatia [3]Department of Neurology, University Hospital Center Zagreb, Kišpatićeva 12, 10000 Zagreb, Croatia [4]Jena University Hospital, Friedrich Schiller University, Institute of Human Genetics, Kollegiengasse 10, D-07743 Jena, Germany

The present case was initially studied due to unexplained cognitive deficit. Physical examination at the age of 18 years revealed cleft palate, lower lip pits and hypodontia, i.e. symptoms characteristic for Van der Woude syndrome (VWS), accompanied with other dysmorphic features and absence of speech. Brain MRI uncovered significantly reduced overall volume of gray matter and cortical gyrification. Banding cytogenetics revealed an indistinct intrachromosomal rearrangement in the long arm of one chromosome 1, and subsequent microarray analyses identified a 5.56 Mb deletion in 1q32.1-1q32.3, encompassing 52 genes; included were the entire IRF6 gene (whose mutations/deletions underlay VWS) and *SRGAP2*, a gene with an important role in neuronal migration during development of cerebral cortex. Besides, a duplication in 3q26.32 (1.9 Mb in size) comprising TBL1XR1 gene was identified. Multicolor banding for

chromosome 1 and molecular cytogenetics applying a battery of locus-specific probes covering 1q32.1 to 1q44 characterized a four breakpoint-insertionalrearrangement-event, resulting in 1q32.1-1q32.3 deletion with a karyotype: 46,XX,der(1)(pter->q32.1::q42.2->q44::q32.3->q42.13::q44->qter),dup(3)(q26.32q26.32).arr[hg19] 1q32.1q32.3(206,279,995-211,840,280)x1,3q26.32(176,738,433-176,929,584)x3. Considering that the human-specific three-fold segmental duplication of SRGAP2 gene evolutionary corresponds to the beginning of neocortical expansion, we hypothesize that aberrations in SRGAP2 are strong candidates underlying specific brain abnormalities, namely reduced volume of grey matter and reduced gyrification. This work was supported in parts by the Croatian Ministry of Science Education and Sport (LB); Business Innovation Croatian Agency – Croatian Institute for Technology BICRO-HIT (FB) and Croatian Academy of Sciences and Arts Foundation and University of Zagreb research support (ZK).

Genetic mechanisms leading to primary amenorrhea in balanced X-autosome translocations

Moysés-Oliveira M.[1], dos Santos Guilherme R.[1,2], Gollo Dantas A.1, Ueta R.[1], Perez A.B.[1], Haidar M.3, Canonaco R.[4], Meloni V.A.[1], Kosyakova N.[2], Liehr T.[2], Carvalheira M.G.[1], Melaragno M.I.[1]

[1]Genetics Division, Department of Morphology and Genetics, Universidade Federal de Sao Paulo, Sao Paulo, Brazil [2]Institute of Human Genetics, Jena University Hospital, Friedrich Schiller University, Jena, Germany [3]Departament of Gynecology, Universidade Federal de

Sao Paulo, Sao Paulo, Brazil [4]Genetics Division, Hospital do Servidor Publico do Estado de Sao Paulo, Sao Paulo, Brazil Here

X-chromosome and autosome breakpoints in women with balanced X-autosome translocations and primary amenorrhea were mapped, with the goal to search candidate genomic loci for female infertility. Three women with balanced X-autosome translocation and primary amenorrhea were included and studied by cytogenetics and array-methods combined with microdissection. All patients presented with breakpoints in the Xq13q21 region. In two patients, the X-chromosome breakpoint disrupted coding sequences of KIAA2022 and ZDHHC15 gene, respectively. Although both gene disruptions caused absence of transcription in peripheral blood, there is no evidence that supports the involvement of these genes with ovarian function. The ZDHHC15 gene belongs to a conserved syntenic region that encompasses the FGF16 gene, which plays a role in female germ line development. The break in the FGF16 syntenic block may have disrupted the interaction between the FGF16 promoter and its cis-regulatory element. In the third patient, although both breakpoints are intergenic, a gene that plays a role in the DAX1 pathway (FHL2 gene) flanks distally the autosome breakpoint. The FHL2 gene may be subject to position effect due to the attachment of an autosome segment in Xq21 region. In conclusion, the etiology of primary amenorrhea in balanced X-autosome translocation patients may underlie more complex mechanisms than interruption of specific X-linked candidate genes, such as position effect. The fine

mapping of the rearrangement breakpoints may be a tool for identifying genetic pathogenic mechanisms for primary amenorrhea. This study was supported by Fundacao de Amparoa Pesquisa do Estado de Sao Paulo (grant 2011/51690-1).

Molecular diagnosis of rare chromosomal and genomic diseases

Iourov I.Y.[1,2,3], Vorsanova S.G.[1,2,4], Yurov Y.B.[1,2,4], Zelenova M.A.[1,2,4], Kurinnaia O.S.[1,2,4]

[1]Mental Health Research Center, Moscow, Russian Federation [2]Research and Clinical Institute for Pediatrics named after Y.E. Veltishev at the Pirogov Russian National Research Medical University, Ministry of Health, Moscow, Russian Federation [3]Department of Medical Genetics, Russian Medical Academy of Postgraduate Education, Ministry of Health, Moscow, Russian Federation [4]Moscow State University of Psychology and Education, Moscow, Russian Federation.

Rare chromosomal and genomic diseases require a sophisticated workflow for the diagnosis and uncovering pathogenic mechanisms for further therapeutic interventions. Genome scan technologies enhanced by bioinformatics allow for association of almost every clinical condition with gene or chromosomal mutations [1, 2]. The data concerning genome variability provides a way to understand the meaning of genetic changes at interindividual and at intercellular levels defining the phenotypic outcome [3]. These data may be regarded as a basis for personalized or genomic medicine by owing to the possibility to determine individual variome profiles for uncovering genetic mechanisms of phenotypic features and endophenotypes. According to own studies using molecular cytogenetic techniques

(i.e. [4, 5]), we have suggested a workflow for molecular diagnosis in rare diseases associated with autism, mental retardation, congenital malformations and epilepsy. Additionally, rare hereditary diseases associated with genome variations represent a challenging issue due to ambiguity of many changes regarding genomic DNA sequence [6]. Application of bioinformatic technologies may solve the problem through assessing a wide range of molecular and cellular processes that cause the disease or its individual symptoms [7]. An updated workflow for molecular diagnosis of rare chromosomal and genomic diseases would represent the following:

1) detailed clinical and phenotypical description for genotype-phenotype correlation;
2) whole genome scan ("variome" analysis);
3) bioinformatics interpretation of whole genome scan data;
4) exome sequencing when no apparent phenotype causing variations are found;
5) bioinformatics analysis of exome sequencing data.

These several steps are able to lead to more effective molecular diagnosis of rare chromosomal and genomic disorders. Positive effect of molecular studies can be achieved through intensive interaction between experts in various medical fields and widespread implementation of genome-wide scan and bioinformatics technologies on the road to propose effective treatment for these presumably incurable diseases [7]. The study was supported by the Russian Science Foundation (project #14-15-00411).

References:

[1] Kloosterman WP, Hochstenbach R. Deciphering the pathogenic consequences of chromosomal

aberrations in human genetic disease. Mol Cytogenet. 2014; 7(1):100.
[2] Smedley D, Robinson PN. Phenotype-driven strategies for exome prioritization of human Mendelian disease genes. Genome Med. 2015; 7(1):81.
[3] Iourov IY, Vorsanova SG, Liehr T, Yurov YB. Aneuploidy in the normal, Alzheimer's disease and ataxia-telangiectasia brain: differential expression and pathological meaning. Neurobiol Dis. 2009; 34(2):212-20.
[4] Vorsanova SG, Iourov IY, Voinova-Ulas VY, Weise A, Monakhov VV, Kolotii AD, Soloviev IV, Novikov PV, Yurov YB, Liehr T. Partial monosomy 7q34-qter and 21pter-q22.13 due to cryptic unbalanced translocation t(7;21) but not monosomy of the whole chromosome 21: a case report plus review of the literature. Mol Cytogenet. 2008; 1:13.
[5] Iourov IY, Vorsanova SG, Kurinnaya OS, Kolotii AD, Demidova IA, Kravets VS, Yurov YB. [The use of molecular cytogenetic and cytogenetic techniques for the diagnosis of Prader-Willi and Angelman syndrome]. Zh Nevrol Psikhiatr Im S S Korsakova. 2014; 114(1):49-53.
[6] Rauch A, Wieczorek D, Graf E, Wieland T, Endele S, Schwarzmayr T, et al. Range of genetic mutations associated with severe non-syndromic sporadic intellectual disability: an exome sequencing study. Lancet. 2012; 380(9854):1674-82.
[7] Iourov IY, Vorsanova SG, Voinova VY, Yurov YB. 3p22.1p21.31 microdeletion identifies CCK as Asperger syndrome candidate gene and shows the way for therapeutic strategies in chromosome imbalances. Mol Cytogenet. 2015; 8:82.

PROCEEDINGS S51

SMARCA2 deletions as a common cause of intellectual disability associated with Nicolaides-Baraitser syndrome

Vorsanova S.G.[1,2,3], Voinova V.Y.[1,2,3], Vasin K.S. [1,2,3], Yurov Y.B.[1,2,3], Iourov I.Y.[1,2,4]

[1]Research and Clinical Institute for Pediatrics named after Y.E. Veltishev at the Pirogov Russian National Research Medical University, Ministry of Health, Moscow, Russian Federation [2]Mental Health Research Center, Moscow, Russian Federation [3]Moscow State University of Psychology and Education, Moscow, Russian Federation [4]Department of Medical Genetics, Russian Medical Academy of Postgraduate Education, Ministry of Health, Moscow, Russian Federation.

Nicolaides Baraitser syndrome (NCBRS, OMIM#601358) is a rare monogenic disorder with recognizable clinical phenotype. Phenotypically, it is characterized by prenatal growth restriction, congenital anomalies (cardiac anomalies, abdominal wall defects, cryptorchidism), microcephaly, short stature, characteristic facial anomalies, distal limb anomalies, sparse hair, moderate/severe intellectual disability, absence of speech, and epileptic seizures. NCBRS is generally the result of intragenic *SMARCA2* mutations. Only two cases of deletions affecting *SMARCA2* were reported in NCBRS patients [1, 2].

Here, we present two cases of NCBRS due to *SMARCA2* deletions detected by molecular karyotyping (resolution: more than 1 kb) and original bioinformatics technology [3, 4]. Deletion sizes were 88008 bp (7 - 27 exons) and 13638 bp (33-34 exons). Patient 1 (24 month old girl) was born prematurely with low birth weight. She demonstrated slow weight gain and hair loss after birth. At 2 years she had partial alopecia, short stature, psychomotor retardation, both inguinal and umbilical hernias, low weight, small head circumference and facial features characteristic for NCBRS. She had pronounced speech delay, but was social and communicable. Seizure activity in the frontal region was detected on EEG. Patient 2 (29 month old boy) was born at 35 weeks with bilateral cryptorchidism. He had feeding problems and sparse hair after birth. At 2,5 years he had low weight, small head circumference and typical for NCRBRS facial features, long toes and fingers with prominent interphalangeal joints. Severe speech impairment was evident along with delay in

motor development. Cardiac anomaly was discovered on sonography. In Patient 1, the whole helicase domain (15-25 exons) of SMARCA2 was lost. All the *SMARCA2* missense mutations and deletions identified so far are clustered in this domain [5]. In Patient 2, the deletion affected two last exons. A number of phosphorylation and acetylation motifs are located in this *SMARCA2* region. Disease-causing deletions of this region have not been previously described.

Mutations in *SMARCA2*, which transcript has an ATP-dependent helicase domain forming a catalytic core of chromatin remodelling SNF2/SWI complex, are supposed to alter transcriptional regulation. According to initial studies NCBRS due to *SMARCA2* mutations was considered an extremely rare genetic condition [5]. However, recent studies have shown that NCBRS is more common in intellectual disability than it was previously recognized [6]. Looking through the Russian cohort (n=324) addressed by the same molecular karyotyping platform and bioinformatics technology [3, 4], we found that *SMARCA2* deletions occur at least in 0.6% cases. This suggests that *SMARCA2* deletions are a common cause of intellectual disability associated with NCBRS. The study was supported by the Russian Science Foundation (project #14-15-00411).

References:

[1] Sousa SB, Hennekam RC; Nicolaides-Baraitser Syndrome International Consortium. Phenotype and genotype in Nicolaides-Baraitser syndrome. Am J Med Genet C Semin Med Genet. 2014; 166C(3):302-14.

[2] Wolff D, Endele S, Azzarello-Burri S, Hoyer J, Zweier M, Schanze I, Schmitt B, Rauch A, Reis A, Zweier C. In-Frame deletion and missense mutations of the C-terminal helicase domain of *SMARCA2* in three patients with Nicolaides-Baraitser syndrome. Mol Syndromol. 2012; 2(6):237-244.

[3] Iourov IY, Vorsanova SG, Korostelev SA, Zelenova MA, Yurov YB. Long

contiguous stretches of homozygosity spanning shortly the imprinted loci are associated with intellectual disability, autism and/or epilepsy. Mol Cytogenet. 2015; 8:77.

[4] Iourov IY, Vorsanova SG, Zelenova MA, Korostelev SA, Yurov YB. Genomic copy number variation affecting genes involved in the cell cycle pathway: implications for somatic mosaicism. Int J Genomics. 2015; 2015:757680.

[5] Van Houdt JK, Nowakowska BA, Sousa SB, van Schaik BD, Seuntjens E, Avonce N, et al. Heterozygous missense mutations in *SMARCA2* cause Nicolaides-Baraitser syndrome. Nat Genet. 2012; 44(4):445-9.

[6] Mari F, Marozza A, Mencarelli MA, Lo Rizzo C, Fallerini C, Dosa L, et al. Coffin-Siris and Nicolaides-Baraitser syndromes are a common well recognizable cause of intellectual disability. Brain Dev. 2015; 37(5):527-36.

PROCEEDINGS S52

The role of immune mechanisms in the pathogenesis of brain diseases

Klushnik T.P., Zozylia S.A

Mental Health Research Center, Moscow, Russian Federation

A lot of recent studies are showing the involvement of immune mechanisms (inflammatory and autoimmune reactions) in the pathophysiology of neuropsychiatric diseases [1, 2]. Immune responses, both in the brain and blood may be induced by different factors (disorders of the immune status of the mother during pregnancy, congenital viral infection, toxins, etc.); in case of a certain genetic predisposition, they contribute to the maintenance of the pathological process in the brain, forming neuroanatomical disorders and behavioral abnormalities, as well as cognitive functions impairments. Presumably, inflammatory factors influence brain neurotransmitter systems through the shifting of tryptophan metabolism towards the

generation of neurotoxic compounds and reciprocal reduction of serotonin levels [2]. Studies demonstrated significant correlations between the activity (the level) of a number of inflammatory factors and clinical features of patients. It is shown that the enzymatic activity of leukocyte elastase, a serine protease which is contained in azurophilic granules of neutrophils and is released into the extracellular space during inflammatory response, is linked to the acuteness and severity of the clinical condition in patients with schizophrenia, affective and autism spectrum disorders, perinatal lesions of the nervous system of various origins. Elevated levels of autoantibodies to neuroantigens are associated with the most severe disorders of the nervous system [3]. However, stronger correlations are detected by using a complex evaluation of the immune system, including both inflammatory and autoimmune markers as a biological indicator [4]. Detection of immune markers in blood, in addition to the fundamental, pathogenic aspect, also has a practical significance due to the fact that they can improve the reliability of the identification of an early diagnosis, to provide laboratory monitoring of disease progression and prognosis. In addition, new methods for management of the nervous system diseases by influencing immune mechanisms may be designed.

References

[1] Kljushnik T.P., Androsova L.V., Simashkova N.V., Zozulja S.A., Otman I.N., Koval'-Zajcev A.A. Sostojanie vrozhdjonnogo i priobretjonnogo immuniteta u detej s psihoticheskimi formami rasstrojstv autisticheskogo spektra. Zh. Nevrologii i psihiatrii im. S.S.Korsakova. 2011; 111 (8): 41-45.

[2] Beumer W., Gibney S.M., Roosmarijn C.D., Pont-Lezica L., Doorduin J., Klein H.C., Steiner J., Connor T.J., Harkin A., Versnel M.A., Drexhage H.A. The immune theory of

psychiatric diseases: a key role for activated microglia and circulating monocytes. J. Leukoc. Biol. 2012; 92:959-975. doi: 10.1189/jlb.0212100.

[3] Bergink V., Gibney S.M., Drexhage H.A. Autoimmunity, inflammation, and psychosis: a search for peripheral markers. Biol Psychiatry. 2014; 75(4):324-31. doi: 10.1016/j.biopsych.2013.09.037.

[4] Androsova L.V., Simashkova N.V., Shushpanova O.V., Otman I.N., Kljushnik T.P. Vozmozhnosti ispol'zovanija nekotoryh immunologicheskih pokazatelej dlja ocenki tjazhesti klinicheskogo sostojanija pacientov s detskoj shizofreniej. Zh.Nevrologii i psihiatrii im. S.S.Korsakova. 2016; 2: 85-89. doi: 10.17116/jnevro20161162185-89

Two cases of cardiofaciocutaneous syndrome without cardiac findings, caused by *BRAF* mutations

Bulatnikova M.A.[1], Vasilishina A.A.[1,2], Kotelevskaya E.A.[1,2], Kuranova M.L.[1], Dvoeglazova M.O.[1], Gumenik E.V.[3], Larionova V.I.[2]

[1]*Stem Cell Bank Pokrovski, Russia* [2]*North-Western State Medical University named after I.I.Mechnikov, Russia* [3]*Medical center "Scandinavia"*

Cardiofaciocutaneous syndrome (CFS) is a rare genetic disorder caused by germline heterozygous mutations of one of four genes: BRAF (about 75% of known cases, MAP2K1, MAP2K2, (about 25%), and KRAS (a few cases). As these four genes encode mitogen activated protein kinase phosphatases of RAS/ERK pathway, CFS have some clinical overlaps with other RASophaties: Noonan syndrome, Costello syndrome, Noonan-like syndrome with loose of anagen hair.

CFS syndrome is characterized by neurological delay, mental retardation, cutaneous abnormalities (hyperkeratosis, xerosis, кeratosis pilaris, erythema, ulerythema opryogenes, café au lait spots, sparse hair), cardiac abnormalities (pulmonic stenosis

and other valvular dysplasias, septal defects, cardiomyopathy) and distinctive craniofacial appearance. As cardiac findings are not always presented and skin findings may be very slight, the right assessment of craniofacial appearance in details become more important for diagnostics.

Two cases of CFS without cardiac manifestation with BRAF mutations are presented below. In Case 2 ulerythema opryogenes was absent, severe growth and weight delay is observed. In result wrong diagnosis Costello syndrome was suspected.

Case 1. Six-year old girl with mental and speech retardation, pure coordination, craniofacial abnormalities and slight skin problems was consulted by geneticist. The high level of impressive speech compare with expressive and friendly disposition was observed. There were not any cases of neurological illness of children in family history. The pregnancy and the delivery were without serious complications, but after delivery pure sucking and severe muscle hypotonia were presented. She was able to sit independently when she was two years old and to walk independently, when she was three. Slight erytema with hyperpigmentation, ulerythema opryogenes and xerosis detected on first month of the life. Eyebrow growth was absent. On the three years old the diagnosis of keratitis follicularis was established by dermatologist. Craniofacial appearance was characterized by high and broad forehead, relative macrocephaly, bitemporal narrowing, ulerythema opryogenes, rare eyebrows, down slanting eyes, deep set eyes, short broad nose, deep philthrum, thin upper lip, broad mandibula, flat face, sparse hair and dysplastic earlobe with broad antihelix.

There were short neck and very broad chest, flat nails. There was congenital hematoma on the left leg. MRI of the brain revealed slight atrophic changes of temporal lobes. No cardiac findings and other pathologic findings was found. Took in attention mental retardation neurological problems, skin abnormalities (distinctive for cardiofaciocutaneus syndrome) and distinctive facial futures, sparse hair and flat nails the diagnosis of cardiofaciocutaneus syndrome without cardiac finding was suspected. Mutation within 14 exon of BRAF (c.1741A>G)(p.Asn581Asp) was found by Sanger sequencing.

Case 2. Four-year old girl with global developmental delay, ataxia with severe muscular hypotonia, growth delay, and epileptic seizures. There was not family history. On 20 week of normal pregnancy the sings of hydrocephalus, intrauterine growth retardation and polyhydramnios was found. Craniofacial appearance was characterized by high and broad forehead, with frontal bossing, bitemporal narrowing, arched eyebrows, down slanting, deep set eyes, hypertelorism, broad nose, deep philthrum, full lips, broad mandibula, flat face, sparse hair and dysplastic earlobes with broad antihelix. We observed short neck and broad chest, long fingers with flat nails. We also found severe Joint hypermobility. Severe xerosis and skin hyperpigmentation with slight erhytema were main skin findings. The diagnosis of Costello syndrome was suspected, cause composition of pathognomonic for Costello syndrome appearance, global developmental delay, ataxia with severe muscular hypotonia, polyhydramnios and intrauterine hydrocephalus. But Sanger

sequencing of all exons of HRAS was not detected any mutations. According to recommendations to exclude cardiofaciocutaneus syndrome in HRAS mutation-negative cases of Costello phenotype and knowledge of many overlaps between CFS and Costello syndrome 6, 11, 12, 13, 15, 16 exons of BRAF was tested by sanger sequencing. The mutation (c.1802A>T)(p.Lys601Ile) in 15 exon was found.

Rare genomic disorders: a high-resolution whole-genome copy number variation (CNV) analysis of children with intellectual disability, congenital malformations and autism

Iourov I.Y.[1,2,3], Vorsanova S.G.[1,2,4], Zelenova M.A.[1,2,4], Vasin K.S.[1,2,4], Kurinnaia O.S.[1,2,4], Shmitova N.S.[1], Ratnikov A.M.[1], Yurov Y.B.[1,2,4]

[1]Mental Health Research Center, Moscow, Russian Federation [2]Research and Clinical Institute for Pediatrics named after Y.E. Veltishev at the Pirogov Russian National Research Medical University, Ministry of Health, Moscow, Russian Federation [3]Department of Medical Genetics, Russian Medical Academy of Postgraduate Education, Ministry of Health, Moscow, Russian Federation [4]Moscow State University of Psychology and Education, Moscow, Russian Federation.

Genomic disorders are conditions caused by (sub)chromosomal or submicroscopic deletions and/or duplications, which result from specific DNA sequence organization predisposing to a genomic rearrangement [1]. Among children with idiopathic neurobehavioral diseases, genomic disorders are commonly diagnosed [2], inasmuch as the majority of them demonstrate extensive clinical heterogeneity and are caused by genomic rearrangements detectable by high-resolution whole-genome

copy number variation (CNV) analysis only [2, 3]. Among children with autistic disorders, this is also the case [4, 5]. Here, we have evaluated the incidence of genomic disease in 237 children with intellectual disability, congenital malformations and autism using whole-genome CNV scan and bioinformatic analysis [5].

CNV associated with genomic disorders were found in 17 (7.2%) out of 237 patients and were associated with the following syndromes: 1p36 microdeletion syndrome, 1p36 microduplication syndrome, 1p32p31 deletion syndrome, 2q23.1 deletion syndrome, 2q37 deletion syndrome, Angelman and Prader-Willi syndromes (deletions at 15q11.2), 5p13 duplication syndrome, 15q13.3 microdeletion syndrome, 15q24 deletion syndrome, 16p11.2 duplication syndrome, 16p11.2 deletion syndrome, 17q21.31 microdeletion syndrome. It should be noted that 16 syndromes were not diagnosed based on phenotypical analysis. In addition to these cases, large-scale CNVs of unascertained clinical significance were found in 45 patients (19%). According to bioinformatics analysis, we speculate that at least some of these aberrations could be of recurrent nature, and have the potential to become a basis for genomic disorders.

Among children with intellectual disability, congenital malformations and autism, the incidence of genomic disorders appears to range between 7.2% and 26%. It is noteworthy that underlying causes for genomic disorders are detected with high resolution whole genome CNV scan [3, 6]. Additionally, these studies allow the definition of phenotypic heterogeneity of genomic disorders and rare copy-

number variants [7], suggesting studying of genomic disorders in idiopathic neurobehavioral diseases of significant importance [2, 5, 7]. To this end, one has to admit that additional studies of contribution of genomic disorders to neurobehavioral phenotypes should be pursued. This study was supported by the Russian Science Foundation (project #14-35-00060).

References:

[1] Carvalho CM, Lupski JR. Mechanisms underlying structural variant formation in genomic disorders. Nat Rev Genet. 2016; 17(4):224-38.

[2] Männik K, Mägi R, Macé A, Cole B, Guyatt AL, Shihab HA, et al. Copy number variations and cognitive phenotypes in unselected populations. JAMA. 2015; 313(20):2044-54.

[3] Vorsanova SG, Yurov YB, Soloviev IV, Iourov IY. Molecular cytogenetic diagnosis and somatic genome variations. Curr Genomics. 2010; 11(6):440-6.

[4] Vorsanova SG, Yurov IY, Demidova IA, Voinova-Ulas VY, Kravets VS, Solov'ev IV, Gorbachevskaya NL, Yurov YB. Variability in the heterochromatin regions of the chromosomes and chromosomal anomalies in children with autism: identification of genetic markers of autistic spectrum disorders. Neurosci Behav Physiol. 2007; 37(6):553-8.

[5] Iourov IY, Vorsanova SG, Kurinnaia OS, Zelenova MA, Silvanovich AP, Yurov YB. Molecular karyotyping by array CGH in a Russian cohort of children with intellectual disability, autism, epilepsy and congenital anomalies. Mol Cytogenet. 2012; 5(1):46.

[6] Iourov IY, Vorsanova SG, Yurov YB. Recent patents on molecular cytogenetics. Recent Pat DNA Gene Seq. 2008; 2(1):6-15.

[7] Girirajan S, Rosenfeld JA, Coe BP, Parikh S, Friedman N, Goldstein A, et al. Phenotypic heterogeneity of genomic disorders and rare copy-number variants. N Engl J Med. 2012; 367(14):1321-31.
